# Phase 2/3 study evaluating safety, immunogenicity, and noninferiority of single booster dose of AVX/COVID-12 vaccine

**DOI:** 10.1126/sciadv.adq2887

**Published:** 2025-06-27

**Authors:** Constantino López-Macías, Martha Torres, Brenda Armenta-Copca, Niels H. Wacher, Arturo Galindo-Fraga, Laura Castro-Castrezana, Andrea Alicia Colli-Domínguez, Edgar Cervantes-Trujano, Isabel Erika Rucker-Joerg, Fernando Lozano-Patiño, Juan José Rivera-Alcocer, Abraham Simón-Campos, Efrén Alberto Sánchez-Campos, Rafael Aguirre-Rivero, Alejandro José Muñiz-Carvajal, Luis del Carpio-Orantes, Francisco Márquez-Díaz, Tania Rivera-Hernández, Alejandro Torres-Flores, Luis Ramírez-Martínez, Georgina Paz-De la Rosa, Oscar Rojas-Martínez, Alejandro Suárez-Martínez, Gustavo Peralta-Sánchez, Claudia Carranza, Esmeralda Juárez, Horacio Zamudio-Meza, Laura E. Carreto-Binaghi, Mercedes Viettri, Damaris Romero-Rodríguez, Andrea Palencia, David Sarfati-Mizrahi, Weina Sun, Héctor Elías Chagoya-Cortés, Felipa Castro-Peralta, Peter Palese, Florian Krammer, Adolfo García-Sastre, Bernardo Lozano-Dubernard

**Affiliations:** ^1^ Unidad de Investigación Médica en Inmunoquímica, UMAE Hospital de Especialidades, Centro Médico Nacional Siglo XXI, Instituto Mexicano del Seguro Social, Ciudad de México (CDMX), México.; ^2^ Laboratorio de Inmunobiología de la Tuberculosis, Instituto Nacional de Enfermedades Respiratorias (INER) “Ismael Cosío Villegas,” CDMX, México.; ^3^ iLS Clinical Research S.C., CDMX, México.; ^4^ Unidad de Investigación Médica en Epidemiología Clínica, UMAE Hospital de Especialidades, Centro Médico Nacional Siglo XXI, Instituto Mexicano del Seguro Social, CDMX, México.; ^5^ Instituto Nacional de Ciencias Médicas y Nutrición Salvador Zubirán (INCMNSZ), CDMX, México.; ^6^ CAIMED Investigación en Salud S.A. de C.V., CDMX, México.; ^7^ Oaxaca Site Management Organization (OSMO) S.C., Oaxaca, México.; ^8^ Centro de Investigación Clínica Acelerada (CICA) S.C., CDMX, México.; ^9^ Clinical Research Institute (CRI) S.C., Edo. de México, Tlalnepantla, México.; ^10^ Centro de Investigación Clínica Chapultepec, CDMX, México.; ^11^ Unidad de Atención Médica e Investigación en Salud (UNAMIS), Mérida, Yucatán, México.; ^12^ Köhler & Milstein Research (K&M) Facultad de Medicina, Universidad Autónoma de Yucatán, Mérida, Yucatán, México.; ^13^ Centro Multidisciplinario para el Desarrollo Especializado de la Investigación Clínica en Yucatán (CEMDEICY) S.C., Mérida, Yucatán, México.; ^14^ Centro de Investigación Clínica del Pacifico (CICPA), Acapulco, Guerrero, México.; ^15^ Red OSMO, Centro de Investigación y Avances Médicos Especializados (CIAME), Cancún, Quintana Roo, México.; ^16^ Instituto Veracruzano de Investigación Clínica (IVIC) S.C., Veracruz, México.; ^17^ Hospital de Cardiología de Aguascalientes, Aguascalientes, México.; ^18^ Investigadores por México, Consejo Nacional de Humanidades, Ciencias y Tecnologías (CONAHCYT), CDMX, México.; ^19^ Posgrado en Inmunología, Escuela Nacional de Ciencias Biológicas, Instituto Politécnico Nacional, CDMX, México.; ^20^ Laboratorio Avi-Mex S.A. de C.V., CDMX, México.; ^21^ Departamento de Investigación en Microbiología, INER “Ismael Cosío Villegas,” CDMX, México.; ^22^ Unidad de Citometría, INER “Ismael Cosío Villegas,” CDMX, México.; ^23^ Department of Microbiology, Icahn School of Medicine at Mount Sinai, New York, NY, USA.; ^24^ Consultora Mextrategy S.A.S. de C.V., CDMX, México.; ^25^ Center for Vaccine Research and Pandemic Preparedness (C-VaRPP), Icahn School of Medicine at Mount Sinai, New York, NY, USA.; ^26^ Department of Pathology, Molecular and Cell-Based Medicine, Icahn School of Medicine at Mount Sinai, New York, NY, USA.; ^27^ Ignaz Semmelweis Institute, Interuniversity Institute for Infection Research, Medical University of Vienna, Vienna, Austria.; ^28^ Department of Medicine, Division of Infectious Diseases, Icahn School of Medicine at Mount Sinai, New York, NY, USA.; ^29^ Global Health and Emerging Pathogens Institute, Icahn School of Medicine at Mount Sinai, New York, NY, USA.; ^30^ The Tisch Cancer Institute, Icahn School of Medicine at Mount Sinai, New York, NY, USA.; ^31^ The Icahn Genomics Institute, Icahn School of Medicine at Mount Sinai, New York, NY, USA.

## Abstract

Low- and middle-income countries face substantial challenges in immunizing against severe acute respiratory syndrome coronavirus 2 (SARS-CoV-2), including high costs, limited access, and insufficient local manufacturing. To address these issues, we developed and locally manufactured the AVX/COVID-12 vaccine using a cost-effective Newcastle disease virus LaSota platform to express a stabilized SARS-CoV-2 spike protein (HexaPro-S). We evaluated the AVX/COVID-12 vaccine in a phase 2/3 parallel-group, double-blind, active-controlled, noninferiority trial with 4056 volunteers, demonstrating its safety, good tolerability, and ability to induce neutralizing antibodies against ancestral SARS-CoV-2 and the Omicron BA.2 and BA.5 variants. It also stimulated interferon-γ–producing CD8^+^ T cells and met the World Health Organization’s noninferiority criteria compared to AZ/ChAdOx-1-S. No vaccinated participants experienced severe disease, hospitalization, or death. These findings support the use of AVX/COVID-12 as a booster to help achieve and maintain population immunity while addressing global inequities in vaccine distribution, and it has been approved for adult booster use in Mexico.

## Teaser

AVX/COVID-12 vaccine is safe, immunogenic, and noninferior to AZ/ChAdOx-1-S in previously vaccinated and/or infected volunteers.

## INTRODUCTION

Severe acute respiratory syndrome coronavirus 2 (SARS-CoV-2) and its variants of concern (VOCs) have caused hundreds of millions of infections and millions of deaths around the globe since 2020 (*1*). At the beginning of 2021, anti–SARS-CoV-2 vaccination campaigns had been launched in almost 200 countries with more than 13.53 billion doses distributed (*2*); as of November 2022, emergency use approval has been granted to 50 vaccine candidates (*3*). These vaccines demonstrated efficacy in phase 3 clinical trials, inducing neutralizing antibodies targeting the spike protein of SARS-CoV-2, among other less-studied antigens (*4*).

Despite the remarkable speed in the development of COVID-19 vaccines since 2020, over 6.9 million official deaths have been attributed to the disease, with excess mortality estimates being three to four times higher (*1*). Although multiple vaccines are available, low- and middle-income countries (LMICs) continue to face challenges such as high costs, limited access, and insufficient local manufacturing capacity, which hinder effective immunization efforts.

While it is true that some high-income countries currently face vaccine surpluses, the issue of inequitable access to vaccines remains critical, particularly in LMICs. This inequity continues to pose substantial barriers to the administration of booster doses, which are essential for preventing new waves of COVID-19 and protecting vulnerable populations such as the elderly, newborns, immunocompromised individuals, and those with comorbidities. Thus, addressing vaccine supply inequity on a global scale remains a pressing issue (*5*).

The AVX/COVID-12 (AVX) vaccine candidate uses the Newcastle disease virus LaSota (NDV-LaSota) strain as a vector to express the HexaPro-S version of the SARS-CoV-2 spike protein, incorporating mRNA that encodes this protein (*6*, *7*). HexaPro-S includes six prolines to stabilize the spike protein in its prefusion conformation, with further modifications such as the introduction of prolines and the removal of the cleavage site between subunits 1 and 2 to render it nonfunctional. The NDV-LaSota strain, widely used as a veterinary vaccine, has proven to be both safe and effective (*8*). Preclinical trials of NDV-based vaccines targeting coronaviruses in poultry and mammals have demonstrated safety and robust immunogenicity (*9*–*11*). NDV’s natural attenuation minimizes risks to humans and birds (*12*), while genetic modifications ensure that the AVX vaccine poses no environmental or agricultural threats (*13*). This is the first phase 3 clinical evaluation of the active NDV vector as a human vaccine platform and the first testing of the SARS-CoV-2 HexaPro-S construct.

One of the main advantages of the NDV-LaSota vector is its standardized production process using embryonated chicken eggs. This process can be easily adapted to existing influenza vaccine manufacturing facilities, enabling the rapid production of large quantities of doses (*14*). As this vaccine is being produced in Mexico, it will substantially boost the country’s capacity to achieve self-sufficiency in COVID-19 vaccine supply. Moreover, this approach can be replicated in other LMICs where the necessary infrastructure and production expertise already exist.

AVX has shown efficacy in preclinical studies, particularly in providing protection to mice and hamsters (*14*, *15*). It has also been demonstrated to be safe and immunogenic in pigs when administered via intramuscular or intranasal routes (*16*).

In the phase 1 clinical trial (NCT04871737), the vaccine was evaluated with various booster regimens administered through both intramuscular and intranasal routes, displaying notable immunogenicity and a favorable safety profile in both cases (*17*). This was further confirmed in a phase 2 clinical trial (NCT05205746), where volunteers who had previously received mRNA, AZ/ChAdOx-1-S (AZ), or inactivated virus vaccines or had been infected with SARS-CoV-2 and exhibited antispike immunoglobulin G (IgG) titers below 1200 U/ml were assessed with AVX as a booster dose. The vaccine demonstrated safety and immunogenicity, inducing neutralizing antibodies against the ancestral SARS-CoV-2 strain and multiple VOCs, including Alpha, Beta, Delta, and Omicron (BA.2 and BA.5) (*18*). While the intranasal administration of the vaccine shows great promise and could revolutionize COVID-19 vaccine delivery by offering a noninvasive route that targets mucosal immunity, initial data indicated that the intramuscular formulation produced higher neutralizing antibody and T cell responses. For this reason, we prioritized the evaluation of the intramuscular route in this phase 2/3 of the clinical trials, ensuring a robust immunogenic response. The intranasal formulation is currently undergoing further optimization to enhance its immunogenicity, and we plan to continue clinical trials with both formulations.

Similar vaccines are currently undergoing clinical trials: an active version in the United States (NCT05181709) and inactivated versions in Vietnam (phase 1, NCT04830800), Thailand (phase 1/2, NCT04764422; phase 3, TCTR20221026004), and Brazil (phase 1, NCT04993209; phase 2/3, NCT05354024) (*19*–*21*).

In this phase 2/3 parallel study, we assessed the safety and immunogenicity of a single intramuscular booster dose of AVX in healthy adults with prior COVID-19 vaccination and/or SARS-CoV-2 infection. We chose the AZ vaccine as the active comparator for this noninferiority immunobridging study due to its widespread use and well-established efficacy. AZ has shown 84.3% effectiveness against asymptomatic SARS-CoV-2 infection and 98.9% effectiveness against COVID-19–related hospitalization (*22*, *23*). Real-world evidence demonstrates the effectiveness of two-dose primary series of AZ against COVID-19, with ≥80% effectiveness against hospitalization due to VOCs including Alpha, Delta, and Omicron (*22*, *23*). It is also the most widely used vaccine globally, administered in 185 countries. More than 79.43 million doses have been given in Mexico since November 2021, including single-dose, full-vaccination, and booster regimens (*24*, *25*). The AVX and AZ vaccines are both vectorized, making AZ an appropriate comparator for this trial. Unlike the previous phase 2 study (*18*), this trial enrolled participants without regard to their baseline antispike antibody levels, which fluctuated because of the ongoing pandemic during recruitment. By doing so, this study addresses the existing research gap regarding the performance of AVX as a heterologous booster and its comparative effectiveness against a widely used vectorized vaccine.

The inclusion of a phase 2 group in the AVX vaccine study is crucial for collecting data under open-population conditions. This phase aims to confirm the vaccine’s safety while assessing its efficacy in comparison to the AZ active comparator, irrespective of baseline antibody levels. Furthermore, the study offers valuable insights into alternative booster strategies, particularly for populations with diverse primary vaccination series across various platforms, including mRNA, adenoviral, inactivated virus, and subunit protein vaccines, regardless of prior SARS-CoV-2 infection.

## RESULTS

### Study population

**Fig. 1.**
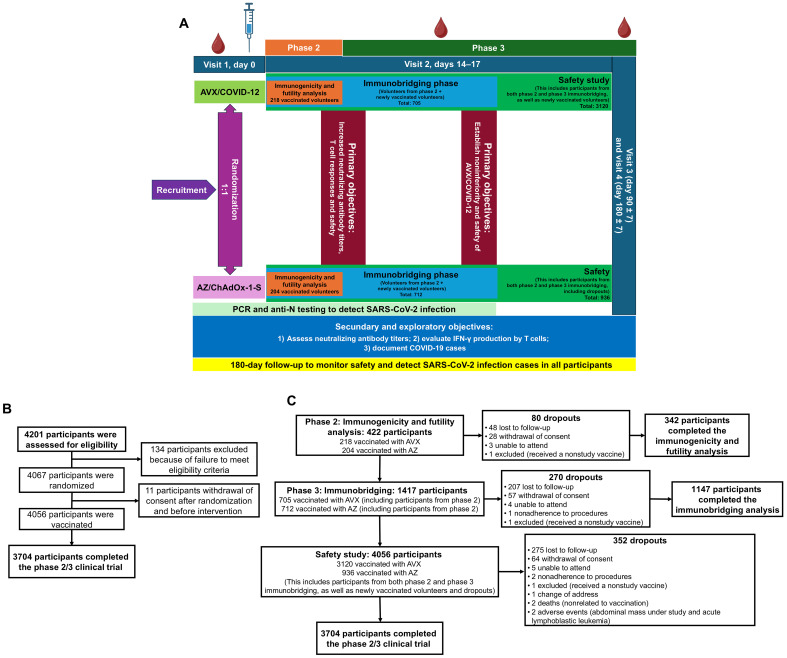
AVX phase 2/3 trial profile. (**A**) Flowchart illustrating the study design and interventions applied to the recruited volunteers. (**B**) Diagram showing the total number of volunteers. (**C**) Allocation of volunteers to experimental groups, along with attrition rates and dropout’s postbooster throughout the study.

A total of 4201 participants were screened for eligibility, and 4067 participants randomized were included into the study. Following randomization, 11 participants withdrew consent, leaving 4056 individuals enrolled for vaccination. In the phase 2 immunogenicity/futility analysis, 422 participants were enrolled—218 received the AVX vaccine, while 204 were administered the AZ vaccine. During this phase, 44 participants from the AVX group and 36 from the AZ group dropped out, resulting in 174 and 168 participants completing the study, respectively (Fig. 1).

In the phase 3 immunobridging trial, a total of 1417 participants were enrolled, with 705 individuals receiving the AVX vaccine (comprising volunteers from phase 2 and newly vaccinated volunteers) and 712 receiving the AZ vaccine (including volunteers from phase 2 and newly vaccinated volunteers). During this phase, 143 participants from the AVX group and 127 from the AZ group dropped out from the study, resulting in 562 participants completing the study in the AVX group and 585 participants in the AZ group (Fig. 1).

For the safety study, volunteers from both the phase 2 immunogenicity/futility and phase 3 immunobridging trials were included, bringing the total to 923 participants in the AVX group and 936 in the AZ group. In addition, volunteers were newly enrolled and vaccinated with AVX, resulting in a total of 3120 participants in this group. Overall, for the safety study, 4056 participants were included, of which 352 dropped out, leaving 3704 who completed the trial (Fig. 1).

### The phase 2 immunogenicity analysis showed that both AVX and AZ booster vaccinations induced neutralizing antibody titers and T cell responses against the ancestral Wuhan-1 strain of SARS-CoV-2

The analysis of neutralizing antibody titers used vesicular stomatitis virus (VSV) expressing the spike protein from the ancestral Wuhan-1 strain. Neutralizing antibody titers in sera from volunteers who received AVX or AZ booster doses were evaluated at days 0 and 14 during the phase 2 immunogenicity/futility analysis. Following booster administration, notable increases in geometric mean (GM) neutralizing titers were observed at day 14 postimmunization, with a *P* value of <0.0001, confirming robust immunogenicity (fig. S1). In addition, the lower limit of the 95% confidence interval (CI) for the GM ratio between AVX and AZ exceeded 0.67, meeting the World Health Organization (WHO) criterion for noninferiority (*26*) and effectively ruling out futility in the study. The results of the futility analysis supported the continuation of the phase 3 immunobridging study.

**Fig. 2.**
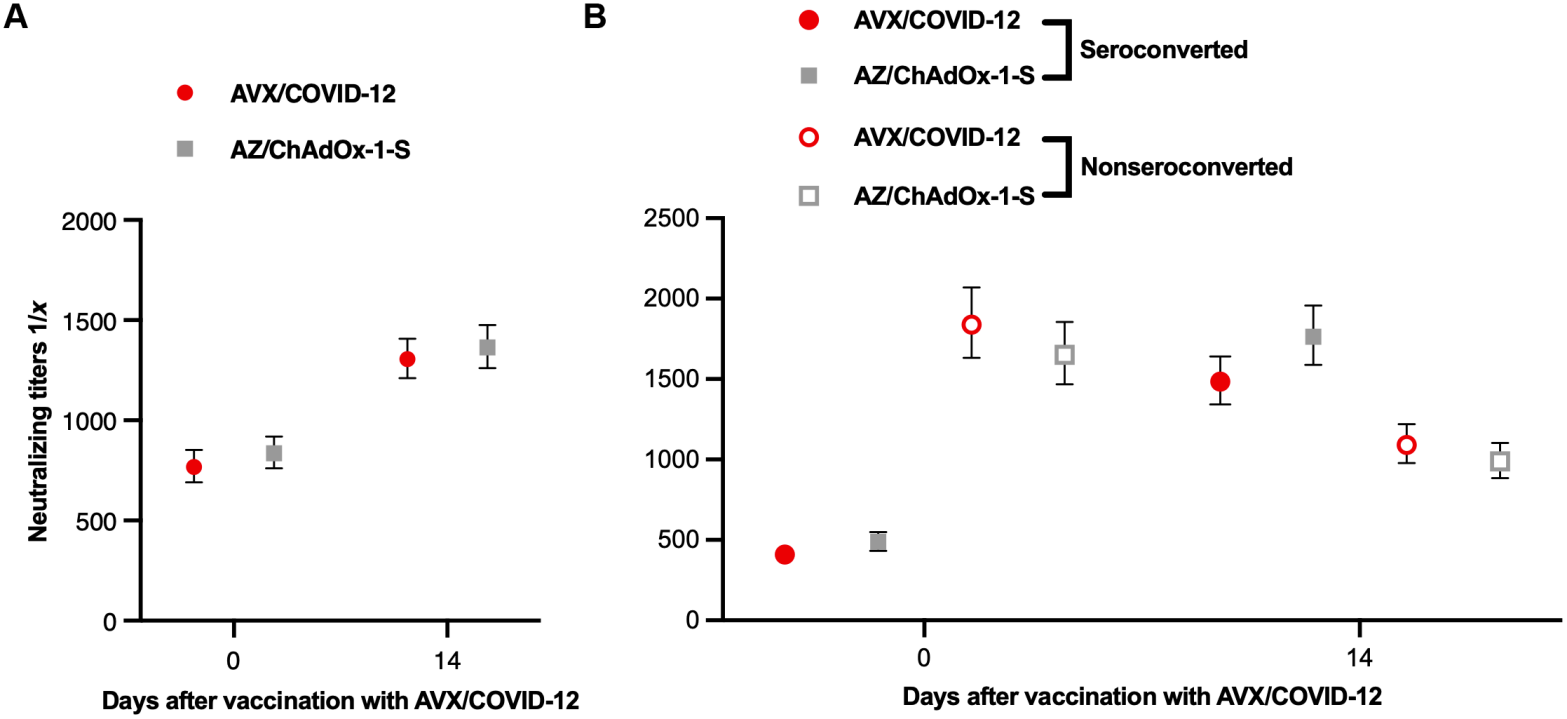
Similar neutralizing antibody responses were observed in participants who received booster doses of AVX or AZ. Analysis of neutralizing antibody titers against the Wuhan-1 strain in participants who received booster doses of AVX (red circles) or AZ (gray squares). (**A**) Neutralizing antibody titers in all boosted participants on days 0 and 14. (**B**) Comparison of antibody titers between participants who seroconverted (closed symbols) and those who did not (open symbols). AVX, *n* = 705; AZ, *n* = 712. The GM ratio and their 95% CI were calculated using an analysis of covariance (ANCOVA) model adjusted for baseline titers, with data transformed to a natural logarithmic scale.

**Fig. 3.**
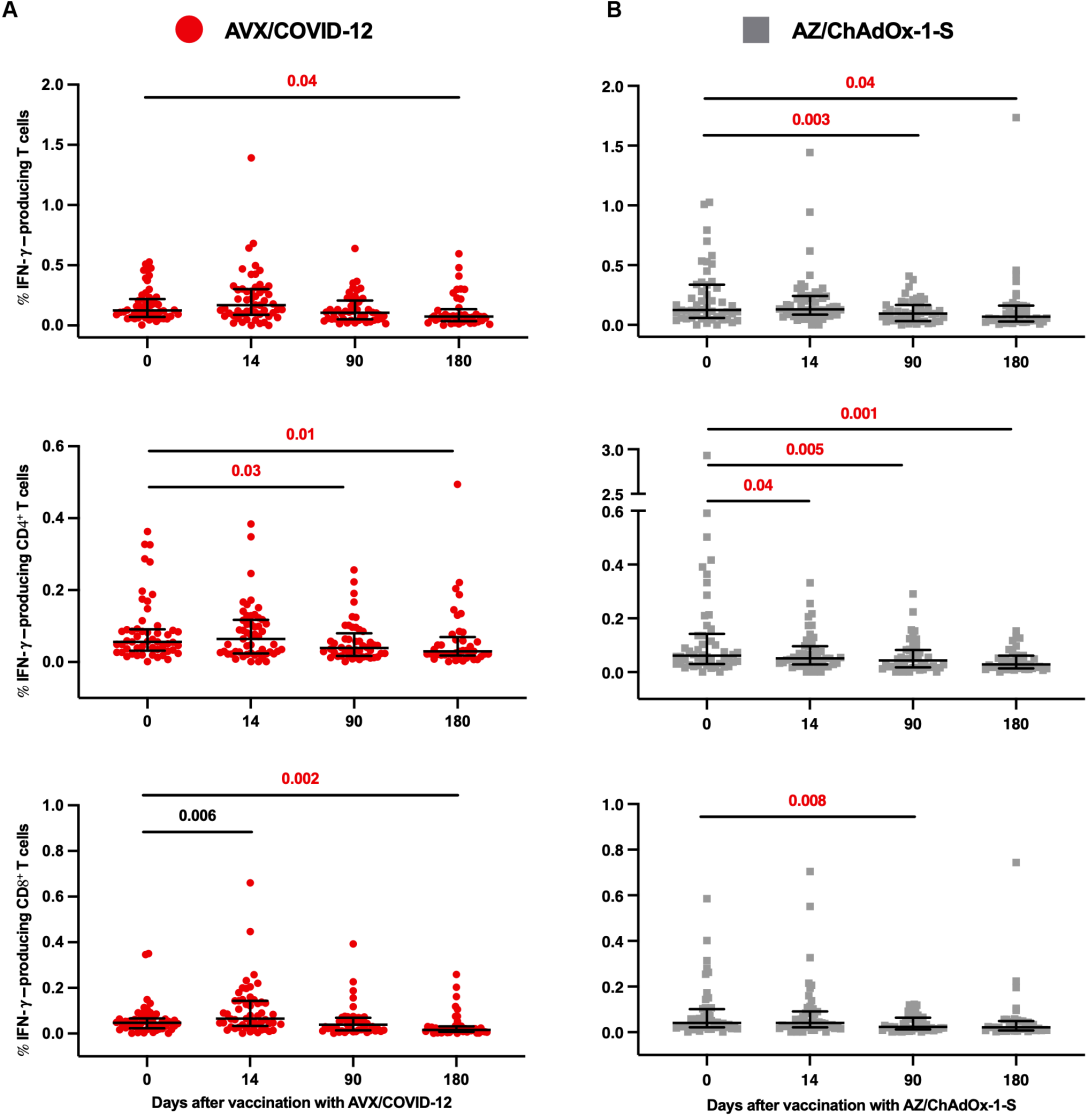
T cell responses in participants vaccinated with AVX or AZ vaccines. (**A**) Percentage of IFN-γ–producing total, CD4^+^, and CD8^+^ T cells from PBMCs collected from participants who received booster doses of AVX or (**B**) AZ vaccine. The cells were stimulated with the subunit 1 of spike protein from the Wuhan ancestral strain, and the production of IFN-γ was assessed using flow cytometry. The limit of detection of the % IFN-γ producing was 0.006, and the graph shows individual values. The bars show GM, and the error shows the 95% CI. Statistically significant increases are annotated in black; this was observed only in IFN-γ–producing T cells from participants vaccinated with AVX. Statistically significant differences with decreases are annotated in red. The sample sizes for AVX were as follows: day 0, *n* = 53; day 14, *n* = 53; day 90, *n* = 44; day 180, *n* = 34. For AZ, the sample sizes were as follows: day 0, *n* = 50; day 14, *n* = 50; day 90, *n* = 40; day 180, *n* = 33. *P* values indicate statistical significance with *P* ≤ 0.05 for intragroup comparisons analyzed using the Wilcoxon signed-rank test.

Figure 2 presents neutralizing antibody titers from participants in the combined phase 2 and phase 3 immunobridging trials. The study assessed the production of interferon-γ (IFN-γ) by peripheral blood total, CD4^+^, and CD8^+^ T cells in response to stimulation with subunit 1 of the spike protein from the ancestral Wuhan-1 strain on days 0 and 14 as a primary objective in phase 2. This analysis used flow cytometry on peripheral blood mononuclear cells (PBMCs) from volunteers who received booster doses of either AVX (Fig. 3A) or AZ (Fig. 3B). In addition, as an exploratory objective, T cell responses in these volunteers were assessed on days 90 and 180 (Fig. 3).

In participants vaccinated with AVX, we observed a slight increase in total T cells and CD4^+^ T cells producing IFN-γ on day 14; however, this rise did not reach statistical significance. A notable increase in the percentage of IFN-γ–producing CD8^+^ T cells (*P* = 0.006) occurred on day 14 compared to baseline levels on day 0 (Fig. 3A and table S1). We observed statistically significant decreases in total IFN-γ–producing T cells only at day 180. For IFN-γ–producing CD4^+^ T cells, a decrease was noted at day 90 with no further decline. The reduction in IFN-γ–producing CD8^+^ T cells was observed only at day 180 (Fig. 3A and table S1).

For volunteers immunized with AZ, no substantial increases were noted in total CD4^+^ and CD8^+^ IFN-γ–producing T cells (Fig. 3B and table S1). Statistically significant decreases in IFN-γ–producing T cells were observed only at days 90 and 180. In the case of the IFN-γ–producing CD4^+^ T cells, the decrease occurred at day 14, with no further drop observed. Last, a reduction in IFN-γ–producing CD8^+^ T cells was observed at day 90, with no subsequent change (Fig. 3B and table S1).

### Phase 3 noninferiority analysis revealed comparable neutralizing antibody responses following booster doses of AVX and AZ vaccines

The noninferiority evaluation of the AVX booster compared to the AZ vaccine included participants from phase 2, along with newly recruited individuals, to achieve target sample sizes of 705 for AVX and 712 for AZ. Both groups were comparable, with no statistically significant differences in age, gender, or race. However, significant differences were noted in participants regarding body mass index (BMI), hypertension (Table 1), and baseline neutralizing antibody titers in seroconverted individuals (Table 2). In addition, prior infection and vaccination history showed no statistically significant differences between the groups (Table 3).

**Table 1.** Demographic characteristics of immunobridging test at baseline. Mann-Whitney *U* tests were conducted to compare for continuous variables, and the chi-square test was used to measure categorical variables. When a *P* value is not displayed, the sample size is inadequate for the analysis. Statistically significant differences are indicated by *P* values: *P* ≤ 0.05.

*n* (%)	AVX	AZ	*P* value
	705 (49.7)	712 (50.3)	
**Age, mean (SD)**	40.3 (14.8)	40.3 (14.5)	0.81
**Age groups (years old)**			
<20	25 (3.5)	28 (3.9)	0.61
20–29	195 (27.6)	191 (26.8)	
30–39	134 (19)	135 (18.9)	
40–49	152 (21.5)	149 (20.9)	
50–59	111 (15.7)	137 (19.2)	
60–69	67 (9.5)	59 (8.2)	
70–79	18 (2.5)	11 (1.5)	
80 and more	3 (0.4)	2 (0.2)	
**Gender, *n* (%)**			
Male	330 (46.8)	341 (47.9)	0.68
Female	375 (53.2)	371 (52.1)	
**BMI, mean (SD)**	27.9 (5.1)	28.8 (5.5)	0.002
**BMI classification**			
Underweight	5 (0.7)	7 (1)	0.008
Normal	214 (30.3)	162 (22.7)	
Overweight	271 (38.4)	285 (40)	
Obesity	215 (30.5)	258 (36.2)	
**Race, *n* (%)**			
Amerindian	13 (1.8)	14 (1.9)	0.71
White	39 (5.5)	50 (7)	
Mexican Mestizo	643 (91.2)	638 (89.6)	
Other	10 (1.4)	10 (1.4)	
**Comorbidities, *n* (%)**			
0	433 (61.4)	380 (53.3)	0.01
1	216 (30.6)	249 (34.9)	
2	43 (6.1)	65 (9.13)	
3 or more	13 (1.8)	18 (2.5)	
**Asthma**	7 (1.0)	8 (1.1)	0.81
**Systolic arterial hypertension**	59 (8.3)	83 (11.6)	0.04
**Type 2 diabetes mellitus**	41 (5.8)	56 (7.8)	0.12

**Table 2.** AVX vaccine meets WHO’s noninferiority criteria in comparison to AZ neutralizing antibody titers at 14 days postboosting. The GM ratio was calculated as GM AVX / GM AZ. The CIs were calculated using Student’s *t* test with the transforming variable on a natural logarithmic scale. The GM ratio and their 95% CIs were calculated using an ANCOVA model adjusted for baseline titers, with data transformed to a natural logarithmic scale.

Baseline				
	AVX	AZ	GM ratio	*P* value
**Participants**	705	712		
**GM**	767.5	836.5	0.92	0.23
**CI**	690.8–852.7	760.7–919.9	0.78–1.03	
**Seroconverted (%)**	410 (58.1)	397 (55.8)		
**GM**	409.5	487.9	0.84	0.05
**CI**	359.9–465.9	433.3–549.3	0.70–1.00	
**Nonseroconverted (%)**	295 (41.9)	315 (44.2)		
**GM**	1837.6	1650.3	1.11	0.20
**CI**	1631.5–2069.7	1467.3–1856.1	0.94–1.31	
**Day 14**				
**Participants**	705	712		
**GM**	1305.2	1364.38	0.96	
**CI**	1210.8–1407.5	1260.7–1476.5	0.85–1.06	
**Seroconverted (%)**	410 (58.2)	397 (55.8)		
**GM**	1484.5	1763.1	0.84	
**CI**	1342.8–1641.1	1587.9–1957.6	0.72–0.97	
**Nonseroconverted (%)**	295 (41.8)	315 (44.2)		
**GM**	1091.5	987.6	1.11	
**CI**	977.2–1,219.2	883.8–1103.6	0.94–1.29	

**Table 3.** History of prior SARS-CoV-2 vaccination and/or infection of participants of the immunobridging test baseline. A clinical history of prior infection was collected during visit 1. Anti-N antibody test was also performed to confirm previous infection in the volunteers. Mann-Whitney *U* tests were conducted to compare for continuous variables, and the chi-square test was used to measure categorical variables. When a *P* value is not displayed (NA), the sample size is inadequate for the analysis. Statistically significant differences are indicated by *P* values: *P* ≤ 0.05.

*n* (%)	AVX	AZ	*P* value
	705 (49.8)	712 (50.2)	
**Clinical history of prior infection, *n* (%)**	33 (4.6)	38 (5.3)	0.57
**Volunteers with positive anti-N antibody test, *n* (%)**	216 (30.63)	229 (32.16)	0.56
**Time (month) since last infection, mean (SD)**	13.7 (7.3)	16 (8.7)	0.28
**Number of prior vaccines, *n* (%)**			
1	218 (30.92)	233 (32.72)	0.73
2	306 (43.40)	294 (41.29)	
3	169 (23.97)	169 (23.73)	
4	12 (1.70)	16 (2.24)	
**Previous vaccination dose(s) received, *n* (%)**			
**AstraZeneca**			
1	182 (25.81)	178 (25)	0.84
2	171 (24.25)	182 (25.56)	
3 or more	45 (6.38)	45 (6.32)	
**Pfizer**			
1	29 (4.11)	31 (4.35)	0.85
2	96 (13.61)	93 (13.06)	
3	3 (0.42)	2 (0.28)	
**CanSino**			
1	118 (16.73)	124 (17.41)	0.24
2	12 (1.70)	7 (0.98)	
**Sputnik**			
1	47 (6.66)	50 (7.02)	0.4
2	42 (5.95)	38 (5.33)	
3	13 (1.84)	8 (1.12)	
**Sinovac**			
1	15 (2.12)	24 (3.37)	0.25
2	28 (3.97)	28 (3.93)	
3	1 (0.14)	0	
**Moderna**			
1	32 (4.53)	23 (3.23)	0.12
2	4 (0.56)	9 (1.26)	
**Novavax**			
1	2 (0.28)	4 (0.56)	0.62
2	4 (0.56)	3 (0.42)	
3	11 (1.56)	18 (2.52)	
**CoronaVac**			
1	3 (0.42)	0	0.99
2	8 (1.13)	3 (0.42)	
**Janssen**			
1	2 (0.28)	3 (0.42)	0.99
2	3 (0.42)	3 (0.42)	
**CureVac**			
1	0	0	
2	0	2 (0.28)	NA

The study conducted the noninferiority assessment through immunobridging by determining the GM of neutralizing antibody titers with a 95% CI, induced by the AVX and AZ vaccines. GM ratios between the two groups were calculated in participants who achieved seroconversion, in accordance with WHO guidelines for noninferiority vaccine trials (*26*).

The analysis of neutralizing antibody titers revealed well-balanced baseline values across the overall participant sample (*P* = 0.23) (Table 2). The GM ratio of neutralizing titers at 14 days between participants immunized with the AVX vaccine and those receiving the AZ vaccine was 0.96 (95% CI, 0.85 to 1.06; Table 2). This finding indicates that AVX is noninferior to AZ (Fig. 2A). Furthermore, this result meets the noninferiority criterion established by the WHO, which specifies that the lower limit of the 95% CI must be greater than or equal to 0.67 (*26*).

Upon further analysis of the neutralizing response, volunteers who received booster doses were stratified into seroconverted (individuals with a greater than twofold increase in preexisting neutralizing antibody titers) and nonseroconverted groups. It was found that 58.15% of individuals in the AVX-vaccinated group seroconverted, compared to 55.75% in the AZ vaccinated group. The AVX-vaccinated group exhibited a 2.4% advantage over the AZ vaccinated group, with a 95% CI of −2.7 to 7.5. This outcome aligns with the noninferiority criterion outlined in the protocol, indicating that the difference in percentages should not exceed −10% (*26*). Notably, individuals in both vaccine groups who experienced seroconversion exhibited an approximately threefold increase in their neutralizing titers 14 days after immunization. In contrast, the nonseroconverted group exhibited lower neutralizing titers on day 14 after the booster for both vaccines (Fig. 2B). This group comprised individuals who had high neutralizing antibody titers before the vaccination boost. These titers remained elevated despite the observed lower neutralizing titers at day 14 (Fig. 2B). We also identified baseline statistical differences in neutralizing antibody titers among seroconverted volunteers (Table 2), as well as differences in BMI and hypertension (Table 1) between the AVX and AZ groups. To further evaluate the impact of these differences, we conducted two analyses of covariance (ANCOVA) (Table 2 and table S2). The results confirmed the noninferiority of AVX compared to AZ, despite the observed differences.

**Fig. 4.**
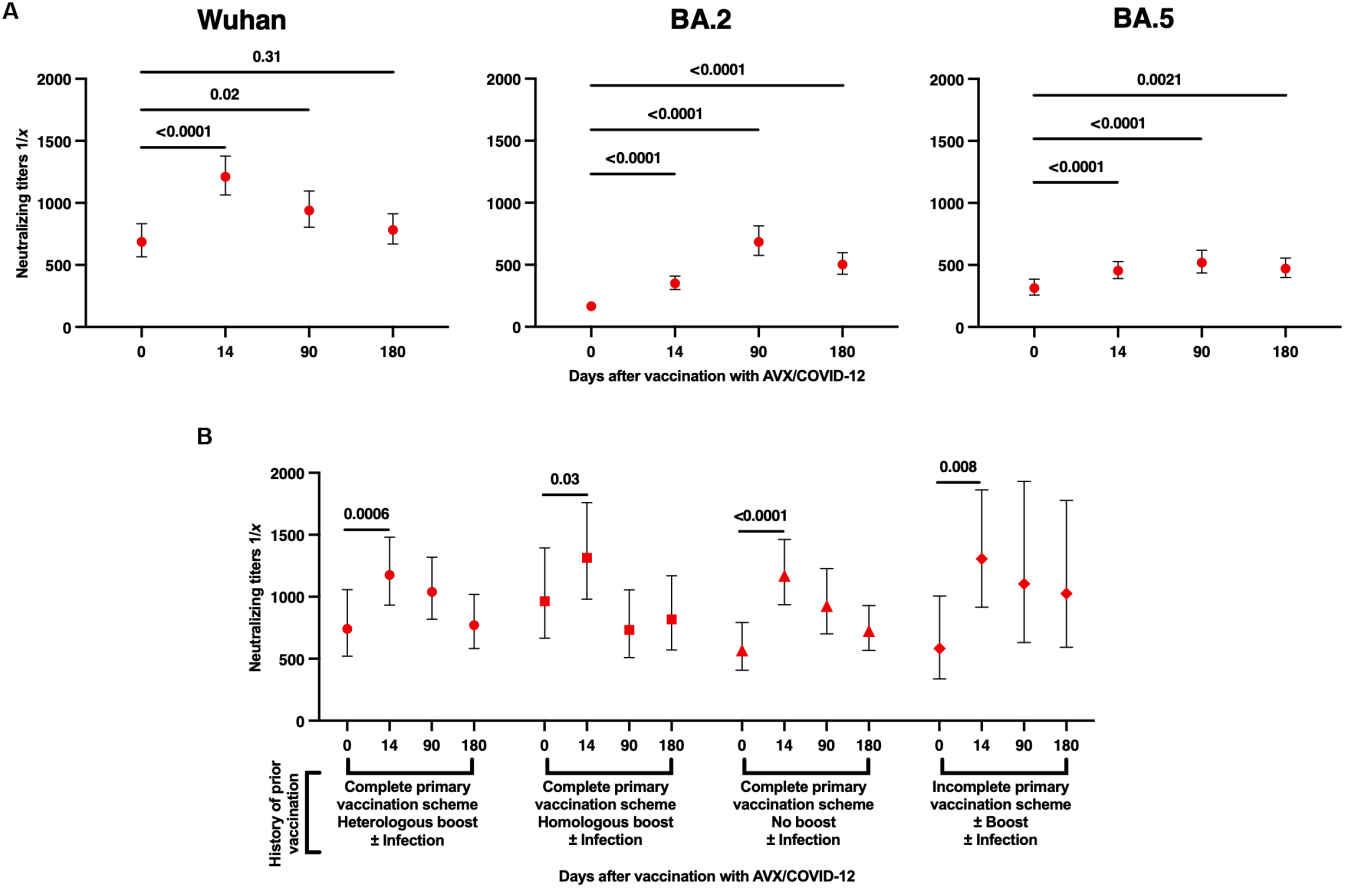
AVX boosting induced neutralizing antibody titers against ancestral Wuhan-1 and Omicron SARS-CoV-2 VOCs. Assessment of neutralizing antibody titers against the spike protein in sera from AVX-boosted volunteers using a pseudovirus neutralization assay. (**A**) Neutralizing antibody titers against Wuhan-1 or Omicron (BA.2 and BA.5) VOCs. (**B**) Specific neutralizing antibody titers against Wuhan-1 strain in participants with different histories of immunization and infection induced by AVX vaccine boost. Homologous boost refers to using the same vaccine platform used for the primary series vaccination; heterologous boost refers to using a different vaccine platform used for the primary series vaccination. ± Infection groups include infected and noninfected participants. The limit of detection was 60, although it is not indicated in the graph. For days 0 and 14, the number of participants was *n* = 218; for day 90, *n* = 195; and for day 180, *n* = 161. The dots in the figures represent the GM, while the bars indicate the 95% CIs. *P* values from the statistical analysis are shown in bold, with *P* ≤ 0.05 considered statistically significant. A paired *t* test was used to compare neutralizing titers.

As exploratory objectives, we assessed neutralizing antibody titers against the Wuhan-1 strain and the Omicron variants (BA.2 and BA.5) in volunteers who were immunized with AVX at 0, 14, 90, and 180 days postimmunization. Titers against the ancestral Wuhan-1 variant substantially increased on day 14, followed by a slight decay on days 90 and 180. In contrast, notably higher neutralizing titers against the BA.2 and BA.5 variants were observed at days 14, 90, and 180 following the booster (Fig. 4A). The pattern of neutralizing titers for BA.2 and BA.5 remained consistent, showing increases at days 14 and 90, followed by a decay at day 180 postboosting (Fig. 4A). However, neutralizing antibody titers against the BA.2 and BA.5 variants were one to three times lower than those against the ancestral Wuhan-1 strain. Despite the decay in titers at day 180, it is important to emphasize that these levels for the BA.2 and BA.5 variants remained substantially higher compared to baseline levels before boosting with the AVX vaccine (Fig. 4A). Further analysis of the total population versus seroconverted individuals revealed an approximate fivefold increase in antibody titers between days 0 and 14 for the seroconverted group, while the total population showed an overall rise of around threefold (fig. S2).

In addition, we analyzed the magnitude of the increase in neutralizing titers at 0, 14, 90, and 180 days following the administration of the AVX vaccine as a single intramuscular booster. This analysis was stratified on the basis of immunization/infection history at the time of recruitment (Fig. 4B). The data consistently demonstrated substantially increased titers at day 14 across all subgroups. However, after this time point, titers tended to decrease in all four subgroups. No differences were noted among the subgroups following the AVX vaccine booster (Fig. 4B). Here, “homologous boost” refers to using the same vaccine platform as the primary series, and “heterologous boost” indicates using a different vaccine platform. The analysis was conducted irrespective of prior infection status (Fig. 4B).

Neutralizing antibody titers following boosting with AVX and AZ were assessed across participants with comorbidities and various age groups. The antibody levels measured at days 0 and 14 indicated that both vaccines similarly enhanced neutralizing antibody titers among participants with comorbidities, as well as those aged over and under 65 years (fig. S3).

Data on prior SARS-CoV-2 infection and vaccination history were collected from participants in the immunobridging test (Table 3). Results show no statistically significant difference in the average time since the last infection and that the proportion of participants with a history of SARS-CoV-2 infection is the same for both groups. Similarly, the proportion of participants vaccinated with different vaccine technologies was also comparable, considering the number of doses received (Table 3).

### Safety study following boosting with AVX

All volunteers from phase 2 and the phase 3 immunobridging study, along with newly vaccinated participants with AVX, were included in the safety study. Among the 4056 participants evaluated for safety, 2111 vaccinated participants reported adverse events (AEs) during the study. The AVX and AZ groups were comparable in age, sex, race, and comorbidities, with both groups having an average age of 39 years (Tables 4 and 5). In the AVX group (*n* = 1603), 51.37% of participants experienced AEs, compared to 54.27% in the AZ group (*n* = 508). This difference was not statistically significant, as shown in table S3.

**Table 4.** Demographic characteristics of participants in the safety trial. Mann-Whitney *U* tests were conducted to compare for continuous variables, and the chi-square test was used to measure categorical variables. When a *P* value is not displayed, the sample size is inadequate for the analysis. Statistically significant differences are indicated by *P* values: *P* ≤ 0.05.

*n* (%)	AVX	AZ	*P* value
	3120	936	
**Age, mean (SD)**	39.8 (15.2)	39.6 (14.6)	0.95
**Age groups (years old)**			
<20	116 (3.7)	43 (4.5)	0.31
20–29	903 (28.9)	264 (28.2)	
30–39	622 (19.9)	180 (19.2)	
40–49	612 (19.6)	189 (20.1)	
50–59	496 (15.9)	163 (17.4)	
60–69	260 (8.3)	78 (8.3)	
70–79	97 (3.1)	16 (1.7)	
80 and more	14 (0.4)	3 (0.3)	
**Gender, *n* (%)**			
Male	1430 (45.8)	440 (47)	0.52
Female	1690 (54.2)	496 (53)	
**BMI, mean (SD)**	28 (5.3)	28.6 (5.5)	0.0006
**BMI classification**			
Underweight	32 (1)	9 (1)	0.001
Normal	966 (31)	228 (24.4)	
Overweight	1168 (37.4)	370 (39.5)	
Obesity	954 (30.5)	329 (35)	
**Race, *n* (%)**			
Amerindian	24 (0.77)	16 (1.7)	0.0001
White	157 (5)	73 (7.8)	
Mexican Mestizo	2928 (93.8)	836 (89.3)	
Other	11 (0.35)	11 (1.1)	
**Comorbidities, *n* (%)**			
0	2665 (85.4)	781 (83.4)	0.07
1	361 (11.6)	128 (13.7)	
2	94 (3)	27 (2.9)	
3 or more	9 (0.28)	7 (0.74)	
**Asthma**	36 (1.15)	12 (1.3)	0.75
**Systolic arterial hypertension**	297 (9.5)	104 (11.1)	0.15
**Type 2 diabetes mellitus**	216 (6.9)	66 (7.1)	0.89

**Table 5.** History of prior SARS-CoV-2 vaccination and/or infection of the participants in the safety trial at baseline. Clinical history of prior infection was collected during visit 1.

*n* (%)	AVX	AZ
	3120	936
**Clinical history of prior infection, *n* (%)**	191 (6.1)	53 (5.6)
**Time (months) since last infection, mean (SD)**	14.2 (10.8)	12.2 (10.9)
**Number of prior vaccines, *n* (%)**		
1	1103 (35.35)	313 (33.44)
2	1324 (42.44)	394 (42.09)
3	600 (19.23)	211 (22.54)
4	87 (2.79)	18 (1.92)
5	6 (0.19)	0
**Previous vaccination dose(s) received, *n* (%)**		
**AstraZeneca**		
1	914 (29.29)	252 (26.92)
2	754 (24.17)	236 (25.21)
3 or more	146 (4.68)	53 (5.66)
**Pfizer**		
1	133 (4.26)	45 (4.81)
2	458 (14.68)	126 (13.46)
3	12 (0.38)	5 (0.53)
**CanSino**		
1	411 (13.17)	172 (18.38)
2	52 (1.67)	10 (1.07)
3	1 (0.03)	0
**Sputnik**		
1	196 (6.28)	50 (5.34)
2	186 (5.96)	44 (4.70)
3	43 (1.38)	9 (0.96)
**Sinovac**		
1	81 (2.60)	28 (2.99)
2	148 (4.74)	34 (3.63)
3	3 (0.10)	0
**Moderna**		
1	78 (2.50)	34 (3.63)
2	38 (1.22)	9 (0.96)
3	2 (0.06)	0
**Novavax**		
1	7 (0.22)	5 (0.53)
2	10 (0.32)	3 (0.32)
3	35 (1.12)	22 (2.35)
**CoronaVac**		
1	8 (0.26)	0
2	20 (0.64)	3 (0.32)
**Janssen**		
1	14 (0.45)	5 (0.53)
2	12 (0.38)	5 (0.53)
**Sinopharm**		
1	2 (0.06)	0
**CureVac**		
1	0	0
2	0	2 (0.21)

**Fig. 5.**
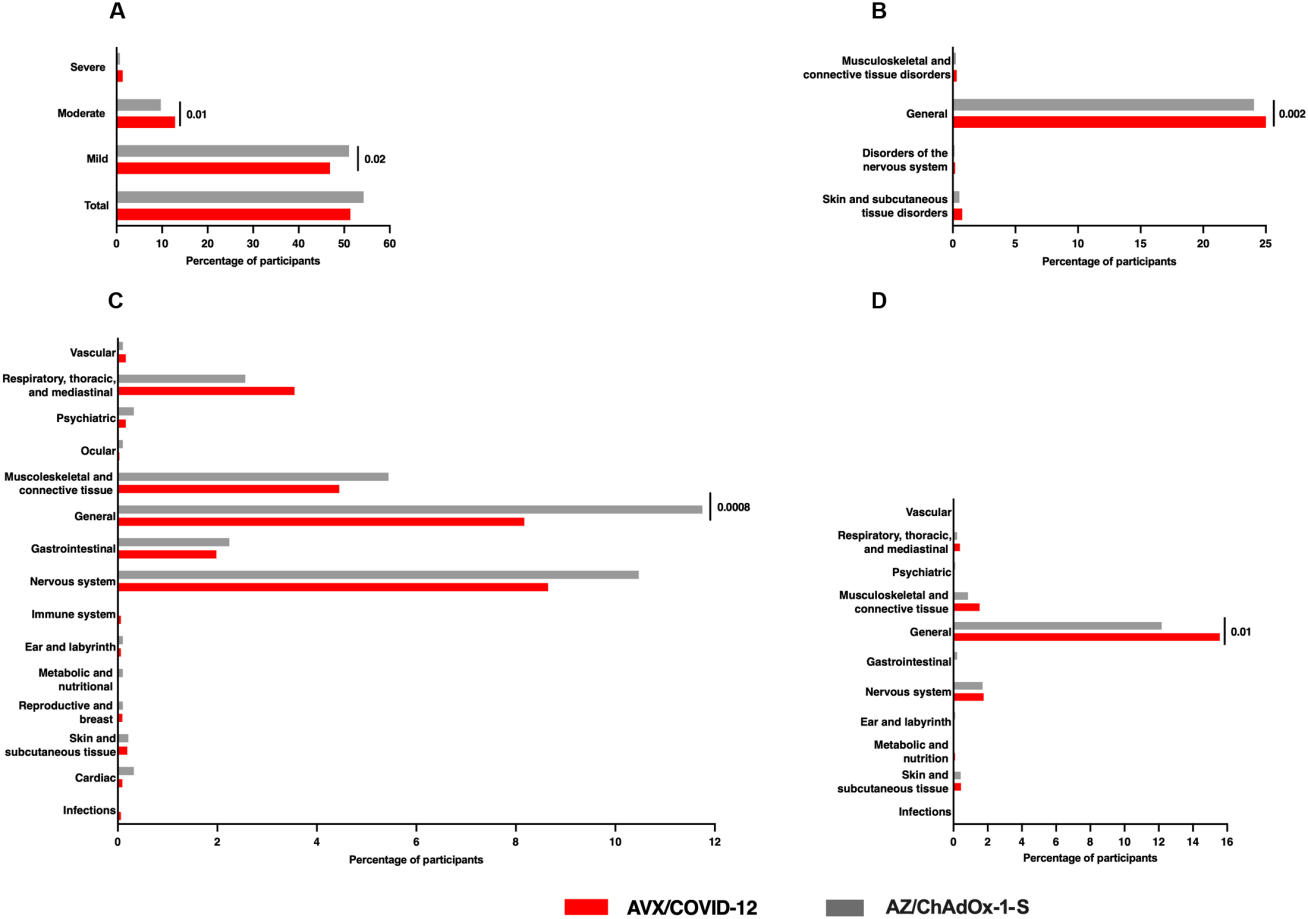
AEs reported within 7 days after boosting with AVX or AZ during the safety phase. AEs reported by participants following a booster dose of either AVX (*n* = 3120) or AZ (*n* = 936) vaccines are presented as follows: (**A**) The percentage of participants experiencing AEs, categorized by severity; (**B**) participants with local AEs of special interest (AESIs); (**C**) participants with systemic AESIs; and (**D**) participants with vaccine-associated AEs (VAAEs) confirmed to be related to the vaccine. “General” refers to disorders affecting the entire organism and those at the immunization site. These disorders, as well as all others depicted in the graphs, are defined in tables S3 to S6. *P* values were calculated using a *z* test for proportions, with statistically significant differences indicated at *P* ≤ 0.05.

When categorizing AEs by severity, a higher proportion of mild events was observed in the AZ group compared to the AVX group (*P* = 0.02). Conversely, for moderate AEs, the proportion was higher in individuals who received the AVX vaccine (*P* = 0.01). Last, no statistically significant differences were found for severe AEs between the two groups (Fig. 5A).

In the context of local AEs of special interest (AESIs), 800 volunteers (25.64%) were documented in the AVX-vaccinated group, while 193 volunteers (20.61%) were recruited in the AZ-vaccinated group. This indicates a statistically significant difference (*P* = 0.002), with a higher incidence observed in the AVX group compared to the AZ group. Regarding musculoskeletal and connective tissue disorders, nervous system disorders, and skin and subcutaneous tissue disorders, there were no statistically significant differences observed between the two groups (Fig. 5B and table S4).

For systemic AESIs within 7 days postvaccination, statistically significant differences were observed only in cases of mild severity. Specifically, 496 cases (15.89%) were reported in volunteers from the AVX group, compared to 183 cases (19.55%) from the AZ group (*P* = 0.008) (table S5). There was a statistically significant difference in general disorders (referring to disorders affecting the entire organism and those at the site of immunization defined in table S5) and administration site alterations in favor of the AVX group (*P* = 0.0008) (Fig. 5C and table S5).

Regarding participants who experienced vaccine-associated AEs (VAAEs), a total of 539 volunteers (17.27%) from AVX and 122 from AZ (13.03%) were reported (table S6). Significant differences (*P* = 0.01) were observed in general disorders (defined in table S6) and administration site alterations (asthenia, pain at the injection site, edema at the injection site, erythema at the injection site, chill, fatigue, hematoma at the injection site, swelling, induration at the injection site, inflammation at the injection site, nerve injury at the injection site, discomfort, general discomfort, fever, and decreased thirst), predominantly occurring in volunteers vaccinated with AVX (Fig. 5D). No statistical differences were observed in the other analyzed disorders.

### Similar incidence of COVID-19 cases in volunteers boosted with AVX or AZ vaccines

As part of the secondary objectives for phase 3, the study documented the occurrence of symptomatic COVID-19 cases in boosted volunteers from both the AVX and AZ groups during the 180 days following administration. A total of 37 COVID-19 cases (5.5%) occurred in the AVX-boosted group, and 42 cases were reported in the AZ group (6.3%), resulting in an incidence rate per 1000 days of 0.29 and 0.32 for AVX and AZ, respectively (table S7). Despite the trial not being designed for efficacy analysis, the log-rank test indicated no significant difference in the cumulative incidence of COVID-19 cases between both groups (*P* = 0.42) (table S7). In addition, a comparison of Nelson-Aalen cumulative hazard incidence curves for COVID-19 cases reported 180 days after boosting with AVX or AZ showed no statistically significant differences between volunteers of these groups (fig. S4).

## DISCUSSION

The development of affordable and effective vaccines, such as AVX using an NDV platform with egg-based manufacturing, offers several advantages, particularly for enhancing self-sufficiency in LMICs. This approach leverages established infrastructure and production processes already in place for influenza vaccines, resulting in a more efficient and cost-effective vaccine production cycle. By using egg-based systems, LMICs can ensure a reliable supply of vaccines tailored to meet local health needs, ultimately bolstering pandemic preparedness and response capabilities. Notably, Mexico, Thailand, and Vietnam have established manufacturing facilities to produce various versions of NDV-based HexaPro-S anti-COVID vaccines. These advancements strengthen local health care systems and contribute to global health security by ensuring equitable access to effective vaccines.

This study is the first phase 3 clinical trial to evaluate the active NDV vector as a human vaccine platform, specifically in combination with the SARS-CoV-2 HexaPro-S construct. The fact that we assessed the impact of a booster dose in healthy adults with high baseline antibody titers mirrors the scenario encountered during current booster campaigns.

The findings from our phase 2 study highlight the AVX vaccine’s robust immunogenicity, evidenced by substantial neutralizing antibody and T cell responses. The induced neutralizing antibody responses met the WHO’s noninferiority criteria (*26*), effectively ruling out futility in the study. This affirmation strengthens our confidence in the vaccine’s potential effectiveness and aligns with our previous results from phase 1 and phase 2 clinical trials (*17*, *18*).

In contrast to the observed antibody responses, no significant changes in T cell responses were noted on day 14 for both the AVX and AZ groups, except the AVX group, which exhibited a significant increase in IFN-γ–producing CD8^+^ T cells. Both groups had high baseline T cell responses before the booster dose. This finding is particularly relevant, as recent studies confirm that vaccination provides protection against both ancestral strains and VOCs, consistently inducing robust CD8^+^ T cell responses in most individuals (*27*). Notably, the proportion of IFN-γ–producing T cells was already relatively high at the start of the study, likely due to the active circulation of the virus in the population, which may have elevated baseline levels of SARS-CoV-2 spike protein–specific T cells (*28*). Alternatively, this could reflect a prolonged antigen-specific T cell response, particularly in CD8^+^ cells, potentially masking the full impact of the booster. If so, then the AVX booster may help sustain hybrid immunity and prolong protection, as seen in cohorts with breakthrough infections, where vaccinated individuals demonstrated more robust cellular responses targeting the spike protein than nonvaccinated controls (*29*).

Reports from 2022 indicated that at least 51% of the population exhibited hybrid immunity, with 85% showing some level of Omicron-induced antibody titers (*30*). In our study, 31.39% of participants demonstrated the presence of anti-nucleocapsid (N) antibodies, indicating previous infection and/or vaccination with an inactivated virus vaccine.

Additional research suggests limited potential for further boosting, particularly in individuals initially primed by infection followed by three doses of mRNA vaccines, which maximally induce spike-specific T cell responses (*31*). This underscores how a history of SARS-CoV-2 infection can modify immune responses to the spike protein, particularly during Omicron infection in vaccinated individuals. Nonetheless, Omicron infections in those who received triple vaccinations generally enhance immune responses to SARS-CoV-2 in both systemic and mucosal compartments, potentially strengthening long-lasting population immunity against the virus (*31*). Furthermore, studies have indicated that vaccination with BNT162b2, based on the Wuhan-1 spike protein, and/or breakthrough infections with early Omicron subvariants elicit CD8^+^ T cell responses that recognize epitopes within the spike protein of newly emerging subvariants (*32*).

The successful induction of these immune responses highlighted the vaccine’s capability to elicit an effective immune response against SARS-CoV-2 and also validates its progression to phase 3 immunobridging studies. Furthermore, these results contribute substantially to the growing body of literature surrounding NDV-based vaccines, reinforcing the potential of this platform for developing effective immunization strategies against infectious diseases.

The phase 3 immunobridging analysis confirms that the AVX vaccine is noninferior to the AZ vaccine, whose efficacy was established through placebo-controlled clinical trials conducted in a naive population during the first pandemic wave in 2020 (*33*). Primary vaccination with the AZ vaccine provides strong protection against COVID-19–related hospitalizations, including those caused by Omicron. Although clinical trials have demonstrated that the AZ booster enhances immunity, real-world data on its effectiveness remain limited. A booster study conducted in England estimated the effectiveness of the AZ vaccine using a test-negative case-control design, reporting 66.1% protection against symptomatic Omicron infection and 82.3% against hospitalization in older adults (*34*). Similarly, a study in Australia analyzed a naive population during the Omicron wave and found that two doses of AZ followed by a heterologous BNT162b2 booster provided 42.0% effectiveness against infection and 81.7% against hospitalization or death (*35*).

However, both studies used a test-negative design, which does not establish causality or constitute randomized evidence. In addition, a meta-regression analysis suggests that the AZ vaccine maintains over 80% effectiveness for at least 6 months postvaccination (*23*). Nonetheless, as an observational study, it is subject to inherent limitations in causal inference, including potential confounding biases. Moreover, it does not account for a substantial non-naive population.

Given that the AZ vaccine was the most widely administered vaccine in Mexico and globally, distributed across 185 countries (*24*, *25*), it serves as an appropriate active control for this noninferiority study. In this phase, the active control was crucial for accurately defining biologically relevant noninferiority and avoiding potential bio-creep (*36*). Although mRNA vaccines may demonstrate higher immunogenicity in some studies, we chose the AZ vaccine as a comparator due to the similarity in their vector-based platforms, making it a suitable reference. The efficacy and effectiveness of the AZ vaccine are well documented, further supporting its selection.

Further data analysis indicated that some individuals in both groups did not seroconvert. Approximately 40% of those vaccinated with AVX and AZ did not seroconvert when measuring response against the ancestral Wuhan-1 strain. The seroconversion observed in volunteers with initially low antibody titers aligns with previous phase 2 clinical trial findings, where participants had similarly low antispike IgG titers at the beginning of the study (*18*). Furthermore, when comparing the overall AVX-vaccinated group to only the seroconverted volunteers, a more pronounced increase in neutralizing titers against the BA.2 and BA.5 variants was observed in the seroconverted group from day 0 to day 14 (fig. S2).

The lack of seroconversion has been reported in infected patients and vaccinated individuals (*37*). This phenomenon is particularly notable among individuals with prior infections, where seroconversion following a vaccine booster is notably lower or even absent when neutralizing antibody titers are elevated (*37*). Our analysis indicates that higher baseline titers are associated with lower seroconversion rates and is consistent with these observations.

In the immunobridging study, 31.39% of volunteers exhibited anti-N protein titers (Table 3), indicating prior infection or vaccination with inactivated vaccines.

Among the volunteers in the AVX and AZ booster groups who did not seroconvert, lower neutralizing antibody titers were observed by day 14. Further analysis revealed that these individuals had very high baseline titers. Notably, this trial included participants regardless of their initial antispike IgG titers. In addition, the study took place during a peak of the Omicron variant outbreak (fig. S5), leading to around 40% of participants having IgG baseline titers of 1024 or higher. This phenomenon has been previously reported in similar cases, especially in volunteers with elevated baseline titers receiving homologous boosters (*37*, *38*).

Research in animal models has shown that immunization schemes involving multiple doses can lead to reduced neutralizing antibody responses, diminished T cell activity, decreased germinal center formation, and cellular response exhaustion (*39*). These findings may help explain the observed responses in our study participants. Furthermore, it is crucial to consider that most of these volunteers had already developed robust hybrid immunity due to prior vaccinations and infections.

These data suggest that boosting does not enhance the immune response in individuals with high neutralizing antibody titers. This information is essential for planning and implementing vaccination campaigns to maximize the benefits of vaccination. These results highlight the effectiveness and importance of timely boosting in specific populations.

In our exploratory analysis, we assessed both antibody and T cell responses at days 90 and 180 postvaccination. As expected, neutralizing antibody levels peaked at day 14 and gradually decayed; however, even at the 6-month mark, antibody titers remained elevated compared to preboost levels, indicating a sustained humoral response. This finding aligns with the durability of immune responses reported for other COVID-19 vaccines (*28*). In contrast, T cell responses showed a steady decay at days 90 and 180, with similar patterns observed across the AVX and AZ vaccine groups. These results suggest that, under the conditions of this trial, the humoral response demonstrates prolonged persistence, while the cellular response decreases more gradually but steadily. This highlights the importance of long-term monitoring of both immune components to fully understand the duration of vaccine-induced protection.

The additional exploratory analysis assessed neutralizing antibody responses against the ancestral Wuhan-1 strain and Omicron BA.2 and BA.5 VOCs. Approximately 40% of participants vaccinated with either AVX or AZ did not seroconvert when measuring responses to Wuhan-1. This figure dropped to 20% when considering participants who did not seroconvert against Wuhan-1, BA.2, and BA.5 (triple negatives). That is, 60% seroconverted against the Wuhan-1 strain, while around 80% seroconverted when responses to Wuhan-1, BA.2, or BA.5 were considered.

Over time, the combination of infection and vaccination generates hybrid immunity, which has been shown to broaden and enhance neutralizing antibody and T cell responses, increasing protection against disease, hospitalization, and death (*40*). This report highlights the induction of a neutralizing response against Omicron VOCs following AVX boosting. Since the vaccine expresses the spike protein of the ancestral Wuhan-1 strain, the anti-Omicron neutralizing response may be attributed to well-documented cross-reactivity (*41*–*43*). This phenomenon is particularly prominent in the context of hybrid immunity, which broadens and enhances neutralizing antibody responses not only against the ancestral strain but also against newer variants, seasonal coronaviruses, SARS-CoV-1, and the Middle East respiratory syndrome coronavirus (*37*).

Given the pandemic conditions during the trial, the induction of antibodies against variants beyond Wuhan-1 is expected, as many T cell and B cell epitopes are shared by the spike proteins of SARS-CoV-2 VOCs (*44*). Collectively, these findings suggest that hybrid immunity, acquired through both vaccination and prior infections, can elicit notably broader and more potent immune responses, potentially offering protection against emerging VOCs.

Moreover, previous studies have demonstrated that HexaPro-S exhibits higher expression levels and improved stability under stress and temperature fluctuations (*7*), which enhances antibody responses (*45*, *46*). Our data confirm the immunogenicity of the HexaPro-S derived from the ancestral Wuhan-1 protein in humans. Notably, most currently available COVID-19 vaccines for human use, contain two proline substitutions to stabilize the spike protein.

The primary focus of COVID-19 booster campaigns has been on elderly individuals, immunocompromised persons, and those with comorbidities. Our exploratory analysis of neutralizing antibody titers following the administration of AVX or AZ boosters in these populations supports the use of AVX as a booster dose for these high-risk groups. Given the host range restrictions, it can be expected that active NDV vaccines could be safe for immunocompromised individuals, pregnant women, and children. However, further research is needed to assess the safety and immunogenicity of NDV-based vaccines in these populations. In addition, administering a second dose and booster with the AVX vaccine is feasible. In our phase 1 clinical trial, we tested AVX as a primary regimen with two doses and a subsequent booster (three doses), observing an increase in neutralizing antibody responses against SARS-CoV-2 after each immunization (*17*). We did not detect a substantial immune response against the NDV vector itself (*18*), confirming that repeat dosing with the NDV-based vaccine is both viable and effective.

For safety study, the assessment of AEs, AESIs, and their VAAEs were assessed in 4056 individuals over 6 months. While using a placebo during the phase 2 stages facilitates a clear definition of the immune response magnitude in individuals receiving the AVX vaccine, incorporating placebo controls, at the time of this trial, presented an ethical conflict, as vaccines with emergency authorization and national vaccination plans became available. Consequently, placebo controls were not used in this study. Instead, AZ was used as an active control to prevent participant distribution bias and serve as a comparator for vaccine safety assessment.

The lack of concerning safety signals among volunteers vaccinated with either AVX or AZ further reinforces the safety profile established in earlier AVX phase 1 and phase 2 trials. This provides added reassurance for the broader application of AVX in vaccination campaigns. These findings are essential for building public confidence in new vaccine candidates and highlight the necessity for ongoing safety monitoring in larger populations, as the vaccine is rolled out in real-world settings.

Although the study was not explicitly designed to evaluate vaccine efficacy, we observed a notable trend indicating that individuals boosted with AVX experienced fewer ambulatory COVID-19 cases than those in the AZ group. The study occurred during an Omicron wave, following a peak in infections (fig. S5), and we did not observe any severe cases or deaths related to COVID-19 in either the AVX or AZ groups. The fact that only mild to moderate cases were noted among participants suggests that AVX vaccination may have conferred a degree of protection to the volunteers.

The ongoing need for safe, effective, and affordable COVID-19 vaccines remains critical, particularly for booster campaigns targeting vulnerable populations and reducing transmission. These findings support using the AVX vaccine to meet this urgent demand. Recently, an inactivated version of this vaccine was authorized for emergency use as a booster to prevent severe COVID-19 in Thailand (*47*), and the AVX vaccine (active) received regulatory approval as a booster in Mexico (*48*).

Our findings demonstrate that the AVX “Patria” vaccine (HexaPro-S) is safe, well tolerated, and highly immunogenic. It effectively stimulated the production of neutralizing antibodies against the ancestral Wuhan-1 strain and the Omicron variants BA.2 and BA.5. In addition, AVX uniquely induced IFN-γ–producing CD8^+^ T cells, further enhancing its immunological profile. The AVX vaccine also meets the WHO’s noninferiority criteria compared to AZ, with a noticeable trend toward a lower incidence of COVID-19 cases in the AVX group. These compelling results strongly support the inclusion of this vaccine as a booster dose for the general population. Its stability, ease of storage, and capacity for large-scale, cost-effective production make it particularly well suited for countries aiming to strengthen domestic vaccine manufacturing capabilities and reduce reliance on external suppliers. Therefore, LMICs, as well as regions with limited vaccine distribution infrastructure, stand to benefit the most from the implementation of the AVX vaccine.

### Limitations

This phase 2/3 trial of the AVX vaccine faced several challenges. A key limitation regarding immunogenicity was the underrepresentation of elderly participants, which may affect the generalizability of our findings to this age group. Future research should prioritize including a larger sample of older adults to better evaluate their immune responses to the AVX vaccine, a critical target for COVID-19 vaccination. The analysis of comorbidities was also constrained by sample size, resulting in only descriptive analyses instead of more robust statistical assessments. We encountered challenges in the exploratory analysis of T cell activation in response to recent VOCs. This analysis was limited by both sample size and the variability in participants’ comorbidities and vaccination regimens, which may have affected the robustness of our findings. Recognizing the importance of exploring CD8^+^ T cell responses, we aim to investigate their implications for long-term immunity in our ongoing research efforts. In addition, the current epidemiological and vaccination landscape within the study population restricts comparisons with a placebo group and precludes the inclusion of unvaccinated individuals or those without prior COVID-19 infection. These limitations highlight the necessity for further studies to investigate the vaccine’s effectiveness and safety across diverse populations and varying health conditions.

## MATERIALS AND METHODS

### Vaccine manufacturing

The AVX vaccine was manufactured under Good Manufacturing Practices at the “Comisión Federal para la Protección contra Riesgos Sanitarios” (COFEPRIS)-approved purpose-built facilities of the Mexican Company Laboratorio Avi-Mex S.A. de C.V. in Mexico City. The vaccine was cultivated in 10-day-old specific pathogen–free chicken embryos through inoculation into the allantoic cavity, using 10^3.3^ 50% egg infectious dose/0.1 ml of the production seed. The embryos were incubated for 72 hours at 37°C with 60 to 70% humidity. After incubation, the embryos were refrigerated for a minimum of 12 hours, and the allantoic fluid (AF) was then aseptically harvested. The AF underwent clarification through filters, was concentrated by a factor of 10× using 300-kDa cassettes, and was subsequently diluted in 20 volumes of phosphate-buffered saline (Lonza, Basel, Switzerland). The resulting AF was frozen and stored at −70°C until needed. The final product was then frozen and stored at −70°C until use. The AVX vaccine was produced as a liquid formulation, supplied in 2-ml vials, and stored frozen. For administration, the intramuscular dose was 0.5 ml.

### Study design

From 9 November 2022 to 4 September 2023, we conducted a phase 2/3 parallel, double-blind, active-controlled, noninferiority, randomized study to evaluate the safety, immunogenicity, and postboost incidence of SARS-CoV-2 infection following a single dose of the AVX vaccine (ClinicalTrials.gov, no. NCT05710783). The study encompassed a phase 2 analysis focusing on immunogenicity and futility, a phase 3 noninferiority assessment via immunobridging that included volunteers from the phase 2 study and a safety evaluation that involved these volunteers along with additional participants to meet the necessary requirements for safety measurements of the AVX vaccine. These phases aimed to establish the immunogenicity of AVX, assess its noninferiority in inducing SARS-CoV-2 neutralizing antibodies 14 days postadministration, and evaluate its safety compared to the AZ vaccine. Participants were 1:1 randomized to receive either a single intramuscular dose of the experimental AVX vaccine at a concentration of 10^8.0^ 50% tissue culture infective dose per dose or a single intramuscular dose of the AZ vaccine at a concentration of 10^10^ viral particles per dose for subsequent noninferiority analysis. The study included four scheduled visits on days 0, 14 (+3 days), 90 (±7 days), and 180 (±7 days), with strict adherence to these windows required. Additional visits were allowed for suspected SARS-CoV-2 infections (Fig. 1).

1) Inclusion criteria: The study recruited adults of both genders aged 18 years or older, who were in good health and had received at least one dose of an approved COVID-19 vaccine. Eligible vaccines included Moderna, Pfizer, AstraZeneca, CanSino, Sinovac, Sinopharm, Johnson & Johnson, and Sputnik V. In addition, participants required a negative polymerase chain reaction (PCR) test for SARS-CoV-2 during the screening visit, a negative pregnancy test for women of childbearing potential, and a commitment to maintaining adequate prevention measures to avoid infection with SARS-CoV-2 throughout the study, particularly during the first 14 days after the baseline visit. There were no restrictions based on baseline antibody titers for eligibility.

2) Exclusion criteria: Adults with a history of hypersensitivity or allergy to any vaccine component, severe anaphylactic reactions, fever at the baseline visit, receipt of the last COVID-19 vaccination within the past 4 months, or SARS-CoV-2 infection within the previous month. Participants who were pregnant or nursing had chronic diseases requiring immunosuppressive agents or immune response modulators, were undergoing active chemotherapy for cancer, had a history of HIV infection, or had chronic renal or liver disease with a recent history of infectious conditions requiring hospitalization or intravenous drug treatment within the year before to the baseline visit.

3) Elimination and substitution criteria: Individuals who withdrew their consent during the study were removed from participation, with data collected up to that point included in the analysis. Those who received additional COVID-19 vaccinations before completing the predefined follow-up periods were also excluded; however, any safety and immunogenicity data collected before to this were analyzed. Elimination criteria also encompassed individuals with major protocol violations, defined as deviations that increased risks or decreased benefits, affecting their rights, safety, or well-being, as well as the integrity of the data, regardless of intent.

Samples from participants in phase 2 and phase 3 immunobridging were analyzed until a negative PCR test result from the baseline visit was confirmed. If an individual was enrolled after testing negative by antigen, was vaccinated, and later tested positive by PCR during the first visit, then they were replaced to meet the calculated sample size. Subsequent samples from these participants were not collected according to the original randomization; however, they were included in the study’s safety population.

Overall, no discontinuations from the study were anticipated for any individual. We performed randomization using a computer-based assignment system, ensuring blinding throughout the study. Upon providing informed consent, each individual was assigned a unique volunteer code that encoded their information in a pseudoanonymized format during data collection and was fully anonymized during data processing and analysis. Participants were randomized in a 1:1 ratio to receive either the AVX or the AZ vaccine. Furthermore, individuals in the initial cohort were additionally randomized to be included in a subsample for assessments of cellular immune responses. In the follow-up safety phase, all participants received the AVX vaccine.

#### Ethics statement

The study was conducted at multiple sites in Mexico, all of which obtained ethics committee approval before to its enrolment (supplemental text S1). In addition, the approved protocol was authorized by the Mexican National Regulatory Authority, the COFEPRIS, under authorization number RNEC2022-AVXSARSCoV2VAC005. The study adhered to Mexican regulations, the Declaration of Helsinki, and Good Clinical Practice principles and was registered in ClinicalTrials.gov (no. NCT05710783). iLS Clinical Research S.C. collaborated with Instituto Mexicano del Seguro Social (IMSS) and Laboratorio Avi-Mex S.A. de C.V. (Avimex) to develop the protocol. All volunteers provided written informed consent.

### Phase 2: Immunogenicity and futility study

Immunogenicity evaluation included assessing neutralizing antibody titers against both ancestral and VOCs of SARS-CoV-2 and the percentage of seroconverted participants. Seroconversion was defined as either a greater than twofold increase in preexisting neutralizing antibody titers against the ancestral Wuhan strain or an increase exceeding two times the lower limit of detection of the assay, measured between prevaccination (day 0) and postvaccination (day 14). In addition, cellular responses induced by the AVX or AZ vaccines were evaluated by measuring the percentage of IFN-γ–producing T cells from PBMCs in a small cohort of randomized volunteers.

An interim futility analysis assessed the feasibility of meeting the noninferiority criterion established for the phase 3 immunobridging study. This analysis included the first participants enrolled and calculated the GM ratio of neutralizing antibody titers and the corresponding 95% CI. A predefined early termination criterion was established comparing GM between the AVX and AZ groups. 

### Phase 3: Noninferiority assay

The study evaluated noninferiority through an immunobridging trial, using the AZ vaccine as the active comparator. According to WHO criteria, noninferiority is established if the ratio of the GM titers of the new vaccine relative to the control is not less than a specified threshold (e.g., 0.67) (*26*). We determined the GM titers ratio of neutralizing antibodies between individuals vaccinated with AVX and those receiving AZ, comparing this ratio to the WHO noninferiority standard to assess whether the AVX vaccine met the criterion.

The Phase 2 immunogenicity and futility study, as well as the Phase 3 noninferiority assay, were conducted following the methods detailed in supplemental text S2.

### Safety study

The safety evaluations in this study focused on the incidence and severity of various types of AEs, including AESIs, all AEs, and VAAEs across both the AZ and AVX vaccine groups. Tables S3 to S6 provide detailed parameters pertaining to these evaluations.

Participants were scheduled for four visits on days 0, 14, 90, and 180, with the option for additional visits if SARS-CoV-2 infection was suspected. To facilitate comprehensive monitoring of AEs, volunteers were instructed to report any AEs occurring postvaccination. They could report these events at any time, either by phone or in person at the clinical center for further evaluation. During the day 14 visit, participants were prompted to recall any AEs experienced since vaccination. Medical staff then categorized and assessed the severity of all reported events. This analysis included participants from the phase 2 and phase 3 immunobridging trials, as well as newly recruited volunteers who received the AVX vaccine. The primary goal was to adhere to WHO guidelines on the clinical evaluation of vaccines, which stipulate a minimum of 3000 individuals must be exposed to the AVX vaccine. Consequently, the safety study included a final safety-only stage, during which only the AVX vaccine was administered until the required number of participants was reached. The comparison with the AZ safety data is for informational purposes only.

### SARS-CoV-2 infection monitoring

SARS-CoV-2 infections during the study were monitored through a combination of methods, including a symptom questionnaire (for suspected or confirmed infections), serological testing for the SARS-CoV-2 N protein, and PCR testing on days 0 and 14 for all volunteers. In addition, individuals with suspected or symptomatic SARS-CoV-2 infection were scheduled for additional visits to confirm infection via molecular diagnosis.

### Primary objectives

Phase 2 was conducted to demonstrate that a single intramuscular administration of the AVX vaccine as a booster dose increases anti–SARS-CoV-2 neutralizing antibody titers starting 14 days postvaccination. Phase 3 was carried out to establish the noninferiority of the AVX vaccine, administered as a single intramuscular booster dose, in terms of the production of anti–SARS-CoV-2 neutralizing antibodies from 14 days postvaccination, compared to the AZ vaccine.

Safety study was performed to demonstrate the safety of a single intramuscular administration of the AVX vaccine as a booster dose starting 14 days postvaccination.

### Secondary objectives

The secondary objectives are as follows: to assess the magnitude of the increase in neutralizing antibody titers at 0, 14, 90, and 180 days after a single intramuscular administration of the AVX vaccine as a booster dose; to evaluate and compare the production of IFN-γ by peripheral blood T cells in response to stimulation with the subunit 1 of spike protein from the Wuhan ancestral strain at 0, 14, 90, and 180 days postadministration of either the AVX or AZ vaccines as a booster dose, within a randomly selected participant subgroup; and to document the incidence of symptomatic and severe COVID-19 cases in both the AVX and AZ vaccine groups starting 14 days after administration.

### Exploratory objectives

The exploratory objectives are as follows: to evaluate the magnitude of the increase in neutralizing antibody titers on days 0, 14, 90, and 180 after a single intramuscular booster dose of the AVX vaccine, considering participants’ immunization and infection history at recruitment; to assess the neutralizing capacity of anti–SARS-CoV-2 antibodies on days 0, 14, 90, and 180 postadministration of the AVX vaccine, using pseudovirus neutralization assays with spike proteins from the ancestral Wuhan strain and Omicron BA.2 and BA.5 variants, in both the overall cohort and those who seroconverted; and to describe the GM titers of neutralizing antibodies following vaccination with AVX and AZ in key subgroups, including individuals younger than 65, those aged 65 and older, and participants with at least one comorbidity, such as obesity (BMI > 30), diabetes mellitus, hypertension, smoking history, or cardiovascular disease.

### Statistical analysis

Descriptive statistics were used to characterize the study population, with measures of central tendency (mean and median) and dispersion (SD and interquartile range) for continuous variables and proportions for categorical variables. The analysis addressed the following hypotheses:

Hypothesis 1: Among individuals previously vaccinated more than 4 months before to the last anti–SARS-CoV-2 dose, at least 80% will exhibit an increase in neutralizing antibody titers compared to baseline, by 14 days postadministration of the AVX vaccine.

Hypothesis 2: The immunogenicity of the AVX vaccine, assessed through neutralizing antibody GM ratios after intramuscular administration, will be noninferior to the AZ vaccine starting 14 days postvaccination.

For the phase 2 immunogenicity and futility analysis, neutralizing antibody titers were compared to baseline levels using paired *t* tests for normally distributed data. An interim futility analysis compared the GM neutralizing titers between the AVX and AZ vaccine groups.

Noninferiority was evaluated by immunobridging in phase 3. The AVX vaccine was considered noninferior if the GM ratio of neutralizing antibody titers was equal to or greater than 1.0, the lower limit of the 95% CI was not less than 0.67, and the lower limit of the CI for the difference in seroresponse rates did not exceed −10% compared to the AZ control.

We applied an ANCOVA model to adjust for baseline differences in neutralizing antibody titers between the AVX and AZ groups, accounting for imbalances in the seroconversion subgroup and other relevant covariates (Table 2 and table S2). We applied the Wilcoxon signed-rank test to assess differences in IFN-γ production by T cells based on data distribution. GM ratios were calculated, and difference of proportion of seroconversion individuals and 95% CIs for the GM ratio noninferiority criteria were calculated using Student’s *t* test on a logarithmic scale and chi-square test for the proportion of seroconversion. Statistical significance was set at *P* ≤ 0.05.

For the safety study, AEs were compared between participants vaccinated with AVX or AZ, considering the severity and relationship to the intervention. AEs proportions were compared using the *z* test. The incidence of symptomatic, severe, or fatal COVID-19 cases was analyzed using Kaplan-Meier survival curves and the Nelson-Aalen cumulative hazard function, with between-group differences assessed using the log-rank test.

Statistical analyses were conducted using STATA v.17.0 (StataCorp, College Station, TX, USA), and GraphPad V10. *P* values are reported in bold, with *P* ≤ 0.05 considered statistically significant.

### Sample size determination

In phase 2, the total sample size was *n* = 400, with 200 participants receiving the AVX vaccine and 200 receiving the AZ vaccine. The sample size was calculated as a subset of the total sample required to meet the immunobridging objectives of phase 3, using the following criteria: Assuming that 60% of the participants recruited will exhibit an inhibitory capacity greater than 95% in a surrogate test, the goal was for at least 80% of the participants receiving the AVX vaccine as a single booster dose to show inhibitory antibody capacity greater than 95% by day 14 postvaccination. A sample of 164 participants was required to detect this effect, based on a *z* test for proportions with an alpha of 0.01 and 90% power. Allowing for a 15% dropout rate, the sample size was increased to 188 participants per group and rounded to 200 for convenience.

The 60% inhibitory capacity estimate was based on expected values from the best available evidence at the time of the surrogate test, which used a 1:20 serum dilution. However, because of fluctuations caused by new infections during waves of VOCs, the actual proportion of participants with inhibitory capacity greater than 95% could have exceeded this estimate. If the baseline proportion was higher, then the test would be repeated with progressively higher dilutions until 60% of participants did not exceed the 95% inhibition threshold. The evaluation at day 14 would then confirm whether at least 80% of participants exhibited inhibitory capacity greater than 95%, ensuring the immunogenicity of the AVX vaccine.

For cellular response analysis, a random sample of 50 participants per group was selected from the 400 participants in phase 2. This sample size assumed a baseline detectable response in 20% of participants at recruitment, increasing to 60% by day 14 postvaccination, based on phase 1 cellular response stimulation results.

In phase 3, the total sample size was *n* = 3832 (3000 receiving the AVX vaccine and 832 receiving the AZ vaccine). We based the calculation on various simulations for the SD of neutralizing titers (on a base-10 logarithmic scale), assuming a GM ratio of 1 and setting the lower bound of the CI at 0.90. We determined the sample size using the following formula for noninferiority studies with a continuous variable of interest

$$n=\frac{2\sigma^{2}{\left(Z_{\propto }+Z_{\beta }\right)}^{2}}{\delta^{2}}$$

where δ represents the difference between the noninferiority limit minus a safety margin. In this case, the noninferiority limit for the GM ratio was set at 1.0, with a safety margin of 0.10, resulting in a lower CI limit of 0.90. The established safety margin was considered feasible because of prior study findings, allowing for an increased sample size to reduce the SD of the variable of interest.

Depending on the analysis of dispersion in neutralizing titers, sample sizes ranged from 142 to 1344 participants per group. We determined the final sample size based on an SD of 0.53 from phase 2 booster studies, using a simulation with an SD of 0.6 to ensure 90% power and an α level of 0.025. We applied a 10% correction factor for potential dropouts, resulting in a final sample size of 832 participants per group.

An additional calculation was conducted for the seroresponse as the variable of interest. Assuming a 96% seroresponse rate (based on interim data from the first 25 participants who received the AVX booster), with a noninferiority margin of 10%, the sample size needed was 252 participants. Thus, the larger sample size of 832 participants per group was deemed sufficient to assess both the neutralizing antibody response and seroresponse with 90% power. To meet safety requirements, a minimum of 3000 participants were planned to be included in the safety population, with 3000 individuals receiving the AVX vaccine and 832 receiving the AZ vaccine.
