# Supplementary material for: Phase 2/3 study evaluating safety, immunogenicity, and noninferiority of single booster dose of AVX/COVID-12 vaccine
Source: Sci Adv. 2025 Jun 27;11(26):eadq2887. doi: 10.1126/sciadv.adq2887 (PMC12204155; doi:10.1126/sciadv.adq2887)
Supplement: Supplementary file 1 — Supplemental text S1 and S2 Figs. S1 to S5 Tables S1 to S7 [file sciadv.adq2887_sm.pdf]

Science Advances
AAAS

Supplementary Materials for

Phase 2/3 study evaluating safety, immunogenicity, and noninferiority of single booster dose of AVX/COVID-12 vaccine

Constantino López-Macías *et al.*

Corresponding author: Bernardo Lozano-Dubernard, lozano@avimex.com.mx;
Constantino López-Macías, constantino.lopez@imss.gob.mx

Sci. Adv. **11**, eadq2887 (2025)
DOI: 10.1126/sciadv.adq2887

This PDF file includes:

Supplemental text S1 and S2
Figs. S1 to S5
Tables S1 to S7

SUPPLEMENTARY MATERIAL

Supplemental text S1. Clinical research sites and corresponding ethics approval numbers in Mexico

Ethics approval for the study was obtained from the Federal Commission for the Protection against Sanitary Risks (COFEPRIS) in Mexico, with the assigned number RNEC2022-AVXSARSCoV2VAC005. As a prerequisite, local ethics clearance was secured from the institutional ethics committees at each participating research site. 1.Research Site: Unidad de Investigacion Medica en Epidemiologia Clinica, UMAE Hospital de Especialidades, Centro Medico Nacional Siglo XXI, Instituto Mexicano del Seguro Social (IMSS), Ciudad de Mexico, Mexico. Ethics Committee: IMSS Scientific Research National Committee. Approval Number: CNIC-2022-785-10. 2.Research Site: Instituto Nacional de Ciencias Medicas y Nutricion Salvador Zubiran, Ciudad de Mexico, Mexico. Ethics Committee: Comite de Etica en Investigacion del Instituto Nacional de Ciencias Medicas y Nutricion Salvador Zubiran. Reference: 4371. 3.Research Site: CAIMED Investigacion en Salud, S.A. de C.V., Ciudad de Mexico, Mexico. Ethics Committee: Comite de Etica en Investigacion de Investigacion Biomedica para el Desarrollo de Farmacos SA de CV. Approval Number: 6030.sEswXEyH. 4.Research Site: Oaxaca Site Management Organization (OSMO) S.C., Oaxaca, Mexico. Ethics Committee: Comite de Etica en Investigacion de Oaxaca Site Management Organization S. C. Approval Number: CEI-OSMO: 1597/2022. 5.Research Site: Centro de Investigacion Clinica Acelerada (CICA), S.C., Ciudad de Mexico, Mexico. Ethics Committee: Comite de Etica en Investigacion de Centro de Investigacion Clinica Acelerada, S.C. Approval Date: 03-sep-22. 6.Research Site: Clinical Research Institute (CRI) S.C., Estado de Mexico, Mexico. Ethics Committee: Comite de Etica en Investigacion Biomedica para el Desarrollo de Farmacos SA de CV. Approval Number: 6389.gjx2xhoih. 7.Research Site: Centro de Investigacion Clinica Chapultepec, Ciudad de Mexico, Mexico. Ethics Committee: Comite de Etica en Investigacion de Sociedad Administradora de Servicios de Salud S.C. Approval Number: 967.hL3XFeyCZscR. 8.Research Site: Unidad de Atencion Medica e Investigacion en Salud (UNAMIS), Yucatan, Mexico. Ethics Committee: Comite de Etica en Investigacion de Unidad de Atencion Medica e Investigacion en Salud. Approval Number: 89244E882AF143D. 9.Research Site: Kohler & Milstein Research (K&M) Facultad de Medicina, Universidad Autonoma de Yucatan, Merida, Yucatan, Mexico. Ethics Committee: Comite de Etica en Investigacion Biomedica para el Desarrollo de Farmacos SA de CV. Approval Number: 6007.MHWWA1. 10.Research Site: Centro Multidisciplinario para el Desarrollo Especializado de la Investigacion Clinica en Yucatan (CEMDEICY) S.C.P, Yucatan, Mexico. Ethics Committee: Comite de Etica en Investigacion Biomedica para el Desarrollo de Farmacos SA de CV. Approval Number: 6007.MHWWA1. 11.Research Site: Centro de Investigacion Clinica del Pacifico (CICPA), Guerrero, Mexico. Ethics Committee: Comite de Etica en Investigacion Biomedica para el Desarrollo de Farmacos SA de CV. Approval Number: 6429.85DQ2ND. 12.Research Site: Red OSMO, Centro de Investigacion y Avances Medicos Especializados (CIAME), Quintana Roo, Mexico. Ethics Committee: Comite de Etica en Investigacion de Oaxaca Site Management Organization S. C. Approval Number: CEI-OSMO: 1732/2022. 13.Research Site: Instituto

Veracruzano de Investigacion Clinica (IVIC) S.C., Veracruz, Mexico. Ethics Committee: Comite de Etica en Investigacion Biomedica para el Desarrollo de Farmacos SA de CV. Approval Number: 6676.UjTJ4KUpK. 14.Research Site: Hospital de Cardiologia Aguascalientes, Aguascalientes, Mexico. Ethics Committee: Comite de Etica en Investigacion de Promotora Medica Aguascalientes S.A. DE C.V. Approval Number: 2394.ceipma.2022. The research was conducted in full compliance with Mexican regulations and in accordance with the principles outlined in the Declaration of Helsinki and Good Clinical Practice

We confirm that all necessary patient/participant consent has been obtained and the appropriate institutional forms have been archived, and that any patient/participant/sample identifiers included were not known to anyone (e.g., hospital staff, patients or participants themselves) outside the research group so cannot be used to identify individuals.

Supplemental text S2 Materials and Methods

Detection of SARS-CoV-2 nucleocapsid protein IgG antibodies

For the detection of IgG against the SARS-CoV-2 nucleocapsid protein, a chemiluminescent microparticle immunoassay (Abbott; Sligo, Ireland) was performed using the Alinity i system. The system calculated the mean chemiluminescence signal from three replicates of the calibrator and stored the result. Results were expressed as the ratio of the sample signal to the calibrator and reported in Index (S/C) units.

For the detection of anti-SARS-CoV-2 nucleocapsid protein antibodies, the LEGEND MAX™ SARS-CoV-2 Nucleocapsid Protein ELISA Kit (BioLegend, San Diego CA, USA) was used following the manufacturer’s instructions. Serum samples were diluted as required and added to pre-coated wells with SARS-CoV-2 nucleocapsid antigen. The plate was incubated at room temperature for a specific duration to allow antibody binding. After incubation, the wells were washed to remove unbound components, and a horseradish peroxidase (HRP)-conjugated detection antibody was added. The plate was incubated again, followed by another washing step to remove excess detection antibody.

A substrate solution was then added, initiating a colorimetric reaction in the presence of bound antibodies. The reaction was stopped by adding a stop solution, and the optical density (OD) was measured at 450 nm using a microplate reader. Antibody concentrations were calculated based on a standard curve generated from the provided controls.

Neutralization assay

The pseudovirus neutralization assay employed pseudovirus particles based on replication-competent vesicular stomatitis virus (VSV)-eGFP-SARS-CoV-2. These particles encoded the spike gene of the ancestral Wuhan-1 strain, as well as Omicron subvariants BA.2 (VSV-SARS-CoV-2 BA.2 clone 1) and BA.5 (VSV-SARS-CoV-2 BA.5 clone 4). The pseudovirus particles were generously provided by Sean Whelan (Washington University, St. Louis, MO, USA) and used in pseudoviral microneutralization assays.

Prior to use in the neutralization assay, all sera were heat-inactivated at 56 °C for 30 minutes. Vero E6 cells (ATCC CRL-1586, Manassas, VA, USA) were seeded at a density of 1.3 x 10^4 cells per well in 96-well plates with 100 µL of complete EMEM (Eagle's minimum essential medium, ATCC, 30-2003 Manassas, VA, USA) supplemented with 10% fetal bovine serum; (FBS; Gibco, Waltham, MA, USA). The cells were then incubated for 24 hours at 37 °C with 5% CO2, allowing them to reach approximately 85% confluence.

The next day, serum samples were diluted in EMEM medium without FBS using a 3-fold serial dilution, starting with a 1:60 dilution. These diluted samples were mixed with 100 TCID50% of VSV-SARS-CoV-2 spike pseudoparticles of the ancestral Wuhan-1 strain or Omicron variants. The mixture was incubated for 60 minutes at 37 °C with 5% CO2 to facilitate the neutralization process. Subsequently, each dilution was transferred to a parallel plate containing Vero E6 monolayers and incubated at 37 °C with 5% CO_2 for 72 hours.

After the incubation period, 3.7% formaldehyde was added to the culture plates and the plates were incubated 30-40 minutes at room-temperature. Then, the formaldehyde was discarded, and the plates were washed twice with PBS (Lonza, Basel, Switzerland). The neutralizing activity of each sample was reported as the titer, represented by the reciprocal of dilution, where the cell protection is 50%; to get this value, we considered the last serum dilution at which the wells are non-infected and with this data, the half maximal 50% inhibitory dilution ($ID_{50\%}$) was calculated using the Spearman-Kaerber analysis method. This analysis was based on the presence or absence of cellular damage (cytopathic effect). Each sample was tested in duplicate.

Is important ensuring that our neutralizing antibody results are comparable to those from other studies, particularly given the variation in readout systems used in vaccine efficacy trials. In our study, we employed a VSV-based pseudovirus microneutralization assay, using cytopathic effect as the readout system. This assay was rigorously validated according to international standards, using secondary reference serum generated in our laboratory and calibrated against the international reference standard (NIBSC: 20/136, assigned value of 250 IU) following WHO guidelines *(47, 48)*.

By incorporating this internationally recognized standard, we ensure that our neutralizing antibody assay produces reliable and comparable results. Our data, expressed as $ID_{50\%}$ (half-maximal inhibitory dilution), adhere to widely accepted practices in neutralization studies, allowing for consistent comparisons with data from other research groups and platforms, such as the Duke assays. While technical specifics may differ between assays, our approach aligns with global standards, facilitating robust cross-study comparisons.

Intracellular cytokine staining assay

PBMCs was isolated from venous blood collected in sodium heparin tubes (BD vacutainer tubes, Franklin Lakes, NJ, USA). Isolation was conducted by density-gradient sedimentation of whole blood diluted at a 1:2 ratio in Roswell Park Memorial Institute (RPMI) 1640 medium (Lonza, Basel, Switzerland) using Lymphoprep (Axis-Shield Diagnostics, Dundee, UK). PBMCs were cryopreserved in a medium consisting of 10% dimethyl sulfoxide

(DMSO) (Sigma Aldrich, St. Louis, MO, USA) and 90% heat-inactivated fetal bovine serum (FBS; GIBCO, California, USA), and stored at -80 °C until use. For T-cell assays, cells were resuspended in CTS OpTmizer (GIBCO, California, USA) to a density of 4 x 10^6 cells/mL. The subunit 1 of the spike protein of SARS-CoV-2 (RayBiotech, Peachtree Corners, GA, USA) was used as the antigen.

Cells were costimulated for 16 hours with anti-human CD28/CD49d (BD Biosciences, San Jose, CA, USA), ancestral Wuhan subunit 1 of the spike protein (RayBiotech, Peachtree Corners, GA, USA) at a concentration of 5 µg/mL. Medium served as a negative control, and phytohemagglutinin (PHA; 1 mg/mL; Burlington, MA, USA) as a positive control. Monensin (GolgiStop BD, San José, CA, USA) was added, and the samples were incubated for 4 hours at 37 °C. After stimulation, the samples were stained with Live/Dead Fixable near (Invitrogen, Waltham, MA, USA) (diluted 1:1000) for 15 minutes in the dark. Subsequently, cell surface staining was performed using a cocktail of anti-human CD3-Alexa Fluor 700 (Biolegend, San Diego, CA, USA), CD4-PerCP/Cy5.5 (Biolegend, San Diego, CA, USA), and CD8-PE/Cy7 (Biolegend, San Diego, CA, USA) antibodies.

The cells were fixed and permeabilized using Cytofix/Cytoperm (BD, San Jose, USA) for 20 minutes. Then, intracellular staining was then performed using a cocktail of anti-human IFN-γ-FITC (BD, San Jose, USA), TNF-α APC (Biolegend, San Diego, CA, USA), and IL-2-BV605 (Biolegend, San Diego, CA, USA) antibodies in Perm/Wash solution for 30 minutes. Unstained and fluorescence minus one (FMO) control were included.

Sample acquisition was carried out on a BD Symphony A1 instrument, previously calibrated with standardized Cytometer Setup and Tracking (CST) beads (BD, San Jose, USA) used for daily quality control. Compensation was performed using CompBeads (BD, San Jose, USA). At least 200,000 events of the lymphocyte region in a Forward Scatter (FSC) vs Side Scatter (SSC) plot were acquired per sample. Analysis was conducted using FACSDiva 8.0.3, and gates for identification of SARS-CoV-2 antigen-specific cytokine-producing $CD3^+$, $CD4^+$, or $CD8^+$ T cells were defined using the FMO controls. This study was approved by the Institutional Review Board for Human Subjects Research at INER.

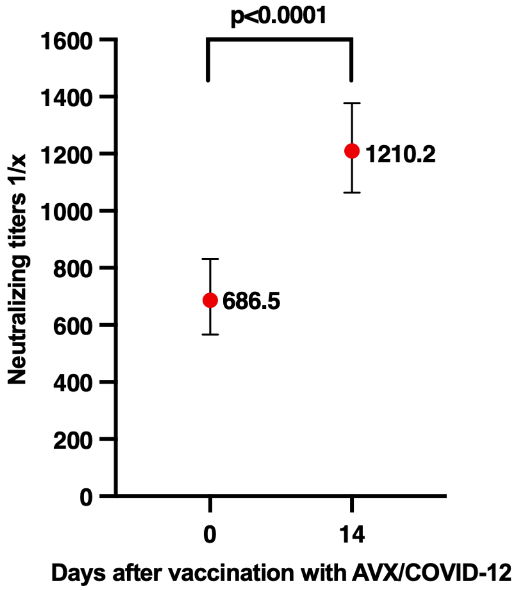

Fig. S1. Seroconversion in subjects receiving the AVX/COVID-12 vaccine. Neutralizing titers against the Wuhan-1 ancestral strain at days 0 and 14 in volunteers from the AVX group. The p-value from the statistical analysis, performed using a paired t-test for neutralizing titers on a natural logarithmic scale, is also shown.

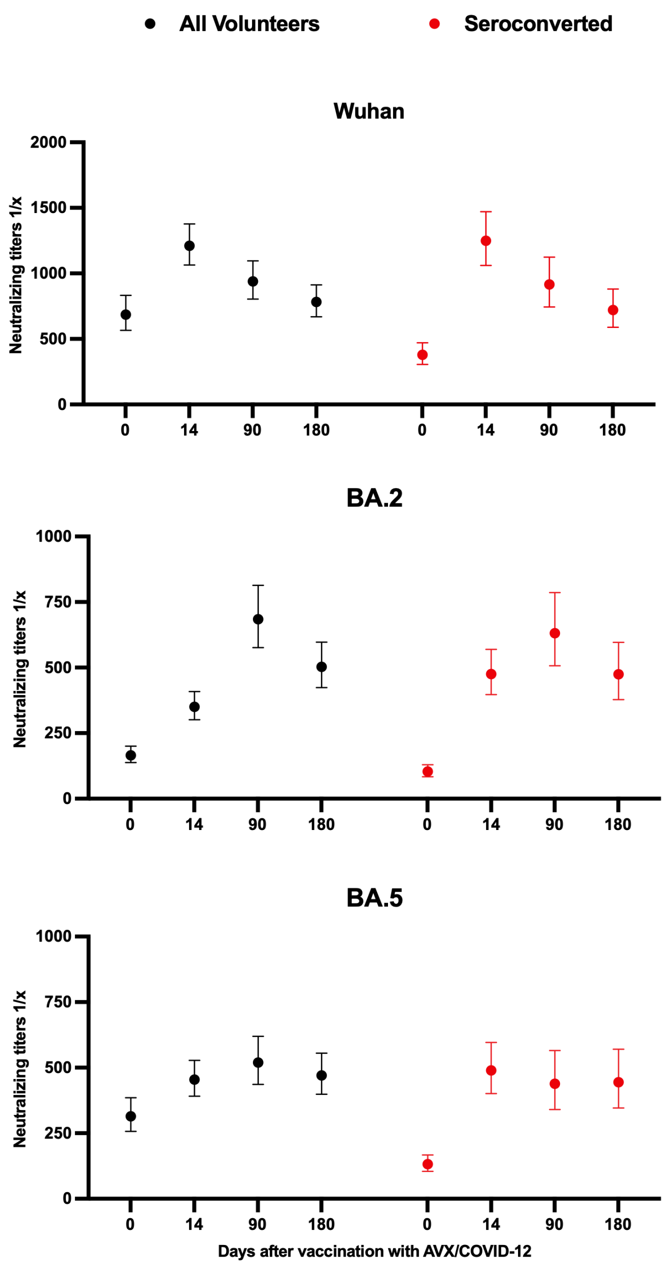

Fig. S2. AVX/COVID-12 boosting induced seroconversion of antibody titers against the ancestral Wuhan-1 and Omicron SARS-CoV-2 variants of concern. Neutralizing antibody titers in sera were measured against the ancestral Wuhan-1 and Omicron variants (BA.2 and BA.5). Black dots represent all volunteers included in this analysis. In contrast, red dots indicate subjects who showed seroconversion. n=218. The dots in the figures represent the GM, and the bars indicate the 95% CIs.

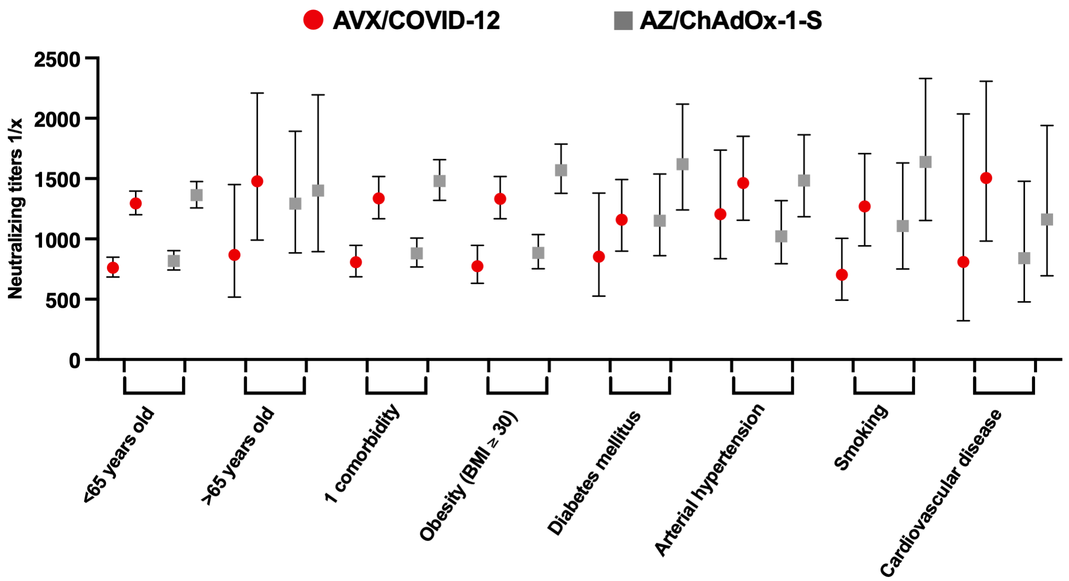

Fig. S3. AVX/COVID-12 and AZ/ChAdOx-1-S boosting elicited comparable increases in neutralizing antibody titers among participants with comorbidities and in subjects >65 and <65 years old. The GM and 95% CIs for antibody titers at day 0 and day 14 are displayed for AVX (red circles) and AZ (gray squares) vaccinated groups. Both vaccines exhibited comparable rises in neutralizing antibody titers among participants with comorbidities and those aged >65 and <65 years old.

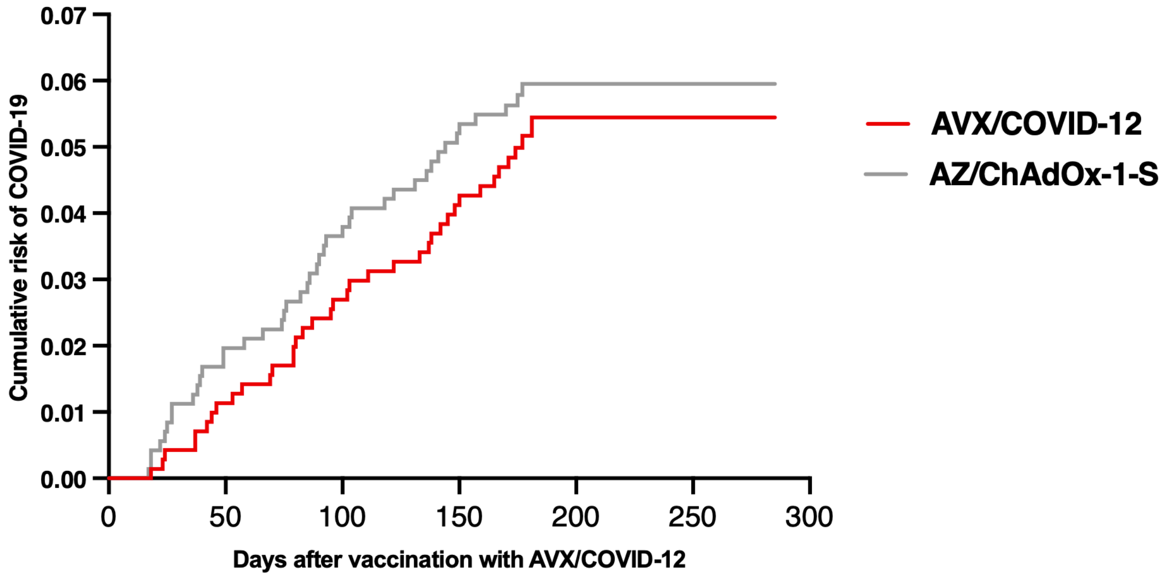

Fig. S4. The incidence of COVID-19 cases showed no statistically significant difference between the participants vaccinated with AVX/COVID-12 or AZ/ChAdOx-1-S. Comparison of Nelson-Aalen cumulative hazard incidence curves for COVID-19 cases reported within 180 days after boosting with AVX or AZ.

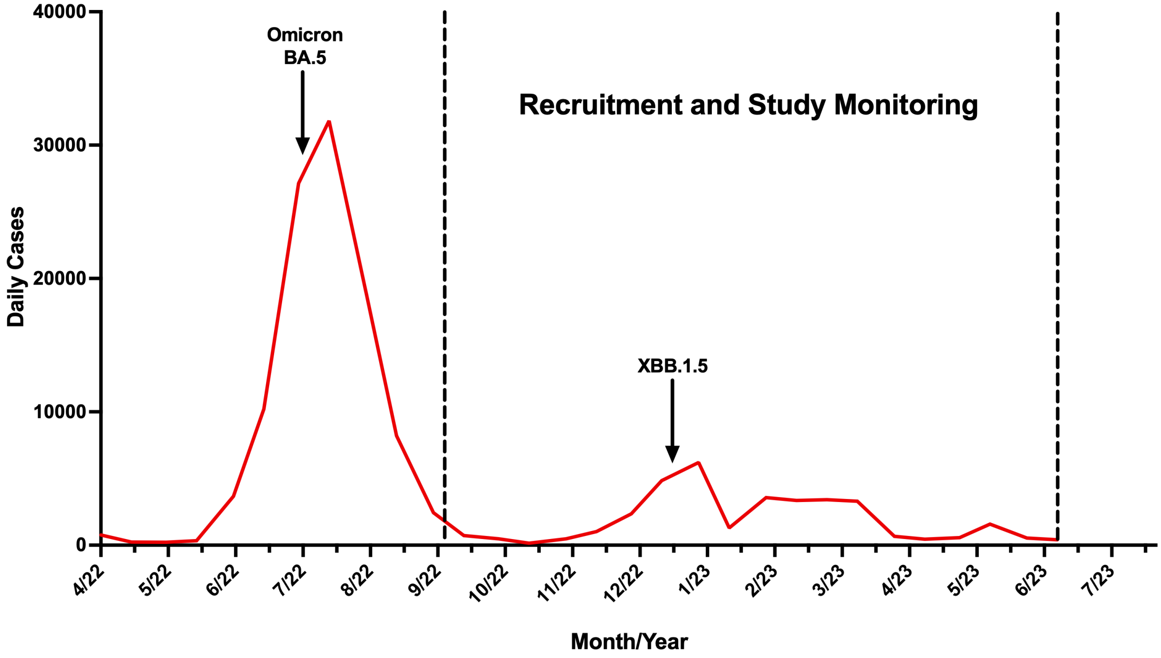

Fig. S5. COVID-19 incidence in Mexico during the Phase II/III clinical trial. This histogram depicts the incidence of COVID-19 cases throughout the recruitment and monitoring phases, represented as daily cases per month reported by the General Directorate of Epidemiology in Mexico. The dotted line denotes the start and end dates of the clinical trial.

Table S1. Percentage of total, CD4+ and CD8+ T-cells producing IFN-γ in response to spike protein stimulation.

Vaccine	AVX/COVID-12				AZ/ChAdOx-1-S			
**Subjects**	**Baseline**<br>53	**Day 14**<br>53	**Day 90**<br>44	**Day 180**<br>34	**Baseline**<br>50	**Day 14**<br>50	**Day 90**<br>40	**Day 180**<br>33
				**T-cells**				
**Median**	0.082	0.076	0.079	0.05 *	0.087	0.098	0.071	0.036 *
**IQR**	0.059 - 0.134	0.059 - 0.152	0.059 - 0.107	0.027 - 0.083	0.066 - 0.129	0.07 - 0.144	0.049 - 0.093	0.021 - 0.077
				**CD4+ T-cells**				
**Median**	0.041	0.051	0.045	0.034	0.05	0.05	0.041	0.0319 *
**IQR**	0.028 - 0.075	0.035 - 0.087	0.029 - 0.062	0.021 - 0.084	0.027 - 0.079	0.033 - 0.075	0.028 - 0.072	0.021 - 0.045
				**CD8+ T-cells**				
**Median**	0.015	0.019	0.017	0.017	0.013	0.021 *	0.016	0.014
**IQR**	0.008 - 0.026	0.011 - 0.033	0.011 - 0.033	0.009 - 0.022	0.006 - 0.033	0.01 - 0.057	0.009 - 0.03	0.006 - 0.023

IQR: Interquartile range p25-p75. P-values: *≤ 0.05 for intra-group comparisons using the Wilcoxon signed-rank test.
* Statistically significant increase compared to baseline values, * Statistically significant decrease compared to baseline values.

Table S2. AVX/COVID-12 vaccine meets WHO non-inferiority criteria compared to AZ/ChAdOx-1-S neutralizing antibody titers at 14 days post-boosting using ANCOVA model

	**Baseline**			
	**AVX/COVID-12**	**AZ/ChAdOx-1-S**	**GM Ratio**	**p-Value**
**Subjects**	705	712		
**GM**	767.5	836.5	0.92	0.23
**CI**	690.8 - 852.7	760.7 - 919.9	0.78 - 1.03	
**Seroconverted**	410	397		
**GM**	409.5	487.9	0.84	0.05
**CI**	359.9 - 465.9	433.3 - 549.3	0.70 - 1.00	
**Non-seroconverted**	295	315		
**GM**	1,837.6	1,650.3	1.11	0.20
**CI**	1,631.5 - 2,069.7	1,467.3 – 1,856.1	0.94 - 1.31	
	**Day 14**			
**Subjects**	705	712		
**GM**	1332.63	1336.66	0.997	
**CI**	1248 - 1422.9	1252.19 - 1426.83	0.90 - 1.09	
**Seroconverted**	410	397		
**GM**	1564.13	1670.5	0.936	
**CI**	1455.3 - 1681.1	1552.4 - 1797.6	0.84 - 1.03	
**Non-seroconverted**	295	315		
**GM**	1046.5	1027.4	1.019	
**CI**	980.2 - 1117.3	864.3 -1094.5	0.93 - 1.11	

Note: GM = Geometric Mean, CI = Confidence Interval. The GM ratio is calculated as GM AVX/COVID-12 / GM AZ/ChAdOx-1-S. CIs were computed using a Student's t-test on a natural logarithmic scale. ANCOVA model with robust errors adjusted for baseline titers, BMI, and hypertension.

Table S3. Proportion of subjects affected by adverse events post-immunization during the 180-day follow-up period.

SOC<br>PT	Severity	AVX	%	AZ	%	Total	%	p-Value
Subjects		3120		936		4056		
Any AE	**Total**	1603	(51.37)	508	(54.27)	2111	(52.04)	0.11
	**MI**	1463	(46.89)	478	(51.06)	1941	(47.85)	0.02
	**Mo**	401	(12.85)	91	(9.72)	492	(12.13)	0.01
	**S**	43	(1.37)	7	(0.74)	50	(1.23)	0.12
Social Circumstances	**Total**	2	(0.06)	0	(0)	2	(0.04)	0.44
	**Mi**	1	(0.03)	0	(0)	1	(0.02)	0.58
	**Mo**	1	(0.03)	0	(0)	1	(0.02)	0.58
	**S**	0	(0)	0	(0)	0	(0)	
Body Modification	Mi	1	(0.03)	0	(0)	1	(0.02)	0.58
Tattoo	Mo	1	(0.03)	0	(0)	1	(0.02)	0.58
Pregnancy, Puerperium, and Perinatal Conditions	**Total**	1	(0.03)	2	(0.21)	3	(0.07)	0.07
	**Mi**	0	(0)	2	(0.21)	2	(0.04)	0.009
	**Mo**	0	(0)	0	(0)	0	(0)	
	**S**	1	(0.03)	0	(0)	1	(0.02)	0.58
Abortion	S	1	(0.03)	0	(0)	1	(0.02)	0.58
Complete Abortion	Mi	0	(0)	1	(0.1)	1	(0.02)	0.07
Pregnancy	Mi	0	(0)	1	(0.1)	1	(0.02)	0.07
Additional Examinations	**Total**	15	(0.48)	4	(0.42)	19	(0.46)	0.83
	**Mi**	12	(0.38)	3	(0.32)	15	(0.36)	0.77
	**Mo**	3	(0.09)	0	(0)	3	(0.07)	0.34
	**S**	0	(0)	1	(0.1)	1	(0.02)	0.07
Arthroscopy	Mo	1	(0.03)	0	(0)	1	(0.02)	0.58
Elevated Blood Pressure	Mi	9	(0.28)	1	(0.1)	10	(0.24)	0.32
Elevated Blood Pressure	Mo	1	(0.03)	0	(0)	1	(0.02)	0.58

SOC PT	Severity	AVX	%	AZ	%	Total	%	p-Value
Increased Respiratory Rate	S	0	(0)	1	(0.1)	1	(0.02)	0.07
Palpable Lymph Node	Mo	1	(0.03)	0	(0)	1	(0.02)	0.58
Increased Weight	Mi	3	(0.09)	0	(0)	3	(0.07)	0.34
Decreased Weight	L	0	(0)	2	(0.21)	2	(0.04)	0.009
Infections and Infestations	**Total**	588	(18.84)	251	(26.81)	839	(20.68)	0.000
	**Mi**	507	(16.25)	238	(25.42)	745	(18.36)	0.000
	**Mo**	101	(3.23)	19	(2.02)	120	(2.95)	0.05
	**S**	2	(0.06)	1	(0.1)	3	(0.07)	0.67
Dental abscess	Mi	1	(0.03)	0	(0)	1	(0.02)	0.58
Dental abscess	Mo	1	(0.03)	0	(0)	1	(0.02)	0.58
Subcutaneous abscess	Mi	0	(0)	1	(0.1)	1	(0.02)	0.07
Appendicitis	Mo	2	(0.06)	0	(0)	2	(0.04)	0.44
Acute appendicitis	Mo	0	(0)	1	(0.1)	1	(0.02)	0.07
Bronchitis	Mi	1	(0.03)	0	(0)	1	(0.02)	0.58
Bronchitis	Mo	2	(0.06)	0	(0)	2	(0.04)	0.44
COVID-19	Mi	191	(6.12)	130	(13.88)	321	(7.91)	0.000
COVID-19	Mo	17	(0.54)	7	(0.74)	24	(0.59)	0.47
COVID-19	S	0	(0)	1	(0.1)	1	(0.02)	0.07
Oral candidiasis	Mo	1	(0.03)	0	(0)	1	(0.02)	0.58
Cystitis	Mo	1	(0.03)	0	(0)	1	(0.02)	0.58
Cystitis	S	1	(0.03)	0	(0)	1	(0.02)	0.58
Dengue	Mo	0	(0)	1	(0.1)	1	(0.02)	0.07
Nail dermatophytosis	Mi	1	(0.03)	0	(0)	1	(0.02)	0.58
Dental pain	Mi	3	(0.09)	0	(0)	3	(0.07)	0.34
Dental pain	Mo	1	(0.03)	0	(0)	1	(0.02)	0.58
Epididymitis	Mi	1	(0.03)	0	(0)	1	(0.02)	0.58
Erysipelas	Mo	1	(0.03)	0	(0)	1	(0.02)	0.58

SOC PT	Severity	AVX	%	AZ	%	Total	%	p-Value
Pharyngitis	Mi	1	(0.03)	0	(0)	1	(0.02)	0.58
Gastroenteritis	Mi	36	(1.15)	12	(1.28)	48	(1.18)	0.75
Gastroenteritis	Mo	15	(0.48)	2	(0.21)	17	(0.41)	0.26
Parasitic Gastroenteritis	Mi	1	(0.03)	0	(0)	1	(0.02)	0.58
Gingivitis	Mi	1	(0.03)	0	(0)	1	(0.02)	0.58
Gingivitis	Mo	0	(0)	1	(0.1)	1	(0.02)	0.07
Hepatitis A	Mi	1	(0.03)	0	(0)	1	(0.02)	0.58
Oral Herpes	Mi	4	(0.12)	1	(0.1)	5	(0.12)	0.87
Oral Herpes	Mo	1	(0.03)	0	(0)	1	(0.02)	0.58
Shingles (Herpes Zoster)	Mi	2	(0.06)	0	(0)	2	(0.04)	0.44
Bacterial Respiratory Tract Infection	Mi	7	(0.22)	0	(0)	7	(0.17)	0.14
Bacterial Respiratory Tract Infection	Mo	1	(0.03)	0	(0)	1	(0.02)	0.58
Skin Infection	Mi	1	(0.03)	0	(0)	1	(0.02)	0.58
Wound Infection	Mi	1	(0.03)	0	(0)	1	(0.02)	0.58
Nail Infection	Mi	1	(0.03)	0	(0)	1	(0.02)	0.58
Upper Respiratory Tract Infection	Mi	244	(7.82)	102	(10.89)	346	(8.53)	0.003
Upper Respiratory Tract Infection	Mo	47	(1.5)	6	(0.64)	53	(1.3)	0.04
Upper Respiratory Tract Infection	S	1	(0.03)	0	(0)	1	(0.02)	0.58
Urinary Tract Infection	Mi	22	(0.7)	6	(0.64)	28	(0.69)	0.83
Urinary Tract Infection	Mo	7	(0.22)	1	(0.1)	8	(0.19)	0.47
Respiratory Tract Infection	Mi	1	(0.03)	0	(0)	1	(0.02)	0.58
Dental Infection	Mi	4	(0.12)	0	(0)	4	(0.09)	0.27
Dental Infection	Mo	1	(0.03)	0	(0)	1	(0.02)	0.58
Vaginal Infection	Mo	3	(0.09)	0	(0)	3	(0.07)	0.34
Herpes Virus Infection	Mi	1	(0.03)	0	(0)	1	(0.02)	0.58
Vulvovaginal Infection by Human Papillomavirus	Mi	0	(0)	1	(0.1)	1	(0.02)	0.07
Influenza	Mi	5	(0.16)	3	(0.32)	8	(0.19)	0.33
Mastitis	Mi	2	(0.06)	0	(0)	2	(0.04)	0.44

SOC PT	Severity	AVX	%	AZ	%	Total	%	p-Value
Onychomycosis	Mi	3	(0.09)	1	(0.1)	4	(0.09)	0.92
Otitis Externa	Mi	1	(0.03)	0	(0)	1	(0.02)	0.58
Otitis Externa	Mo	1	(0.03)	0	(0)	1	(0.02)	0.58
Acute Otitis Media	Mi	2	(0.06)	3	(0.32)	5	(0.12)	0.05
Acute Otitis Media	Mo	4	(0.12)	0	(0)	4	(0.09)	0.27
Pericoronitis	Mo	1	(0.03)	0	(0)	1	(0.02)	0.58
Rhinitis	Mi	7	(0.22)	1	(0.1)	8	(0.19)	0.47
Rhinitis	Mo	1	(0.03)	0	(0)	1	(0.02)	0.58
Sinusitis	Mi	1	(0.03)	0	(0)	1	(0.02)	0.58
Bacterial Sinusitis	Mi	2	(0.06)	0	(0)	2	(0.04)	0.44
Bacterial Sinusitis	Mo	1	(0.03)	0	(0)	1	(0.02)	0.58
Post-Acute COVID-19 Syndrome	Mi	3	(0.09)	1	(0.1)	4	(0.09)	0.92
Scalp Ringworm	Mi	1	(0.03)	0	(0)	1	(0.02)	0.58
Bacterial Tonsillitis	Mi	2	(0.06)	0	(0)	2	(0.04)	0.44
Whooping Cough	Mi	0	(0)	1	(0.1)	1	(0.02)	0.07
Whooping Cough	Mo	0	(0)	1	(0.1)	1	(0.02)	0.07
Vector-Borne Infection Transmission	Mi	2	(0.06)	0	(0)	2	(0.04)	0.44
Tuberculosis	Mo	1	(0.03)	0	(0)	1	(0.02)	0.58
Chickenpox	Mi	1	(0.03)	0	(0)	1	(0.02)	0.58
Chickenpox	Mo	1	(0.03)	0	(0)	1	(0.02)	0.58
Benign, Malignant, and Unspecified Neoplasms (including Cysts and Polyps)	**Total**	2	(0.06)	2	(0.21)	4	(0.09)	0.20
	**Mi**	1	(0.03)	2	(0.21)	3	(0.07)	0.07
	**Mo**	0	(0)	0	(0)	0	(0)	
	**S**	1	(0.03)	0	(0)	1	(0.02)	0.58
Ovarian Adenoma	Mi	0	(0)	1	(0.1)	1	(0.02)	0.07
Uterine Leiomyoma	Mi	0	(0)	1	(0.1)	1	(0.02)	0.07
Intraductal Proliferative Breast Lesion	Mi	1	(0.03)	0	(0)	1	(0.02)	0.58

SOC<br>PT	Severity	AVX	%	AZ	%	Total	%	p-Value
Acute B-Cell Precursor Leukemia	S	1	(0.03)	0	(0)	1	(0.02)	0.58
Benign Ovarian Tumor	Mi	0	(0)	1	(0.1)	1	(0.02)	0.07
Traumatic Injuries, Intoxications, and Complications of Therapeutic Procedures	**Total**	27	(0.86)	6	(0.64)	33	(0.81)	0.50
	**Mi**	18	(0.57)	4	(0.42)	22	(0.54)	0.58
	**Mo**	5	(0.16)	2	(0.21)	7	(0.17)	0.72
	**S**	4	(0.12)	0	(0)	4	(0.09)	0.27
Skin Abrasion	Mi	2	(0.06)	0	(0)	2	(0.04)	0.44
Road Traffic Accident	Mi	2	(0.06)	0	(0)	2	(0.04)	0.44
Road Traffic Accident	Mo	1	(0.03)	0	(0)	1	(0.02)	0.58
Asphyxia	S	1	(0.03)	0	(0)	1	(0.02)	0.58
Fall	Mi	0	(0)	1	(0.1)	1	(0.02)	0.07
Clavicle Fracture	Mo	0	(0)	1	(0.1)	1	(0.02)	0.07
Rib Fracture	Mi	1	(0.03)	0	(0)	1	(0.02)	0.58
Foot Fracture	Mi	2	(0.06)	0	(0)	2	(0.04)	0.44
Hand Fracture	Mo	0	(0)	1	(0.1)	1	(0.02)	0.07
Dental Fracture	Mi	1	(0.03)	0	(0)	1	(0.02)	0.58
Multiple Fractures	Mi	1	(0.03)	0	(0)	1	(0.02)	0.58
Olecranon Fracture	S	1	(0.03)	0	(0)	1	(0.02)	0.58
Heat Stroke	Mi	1	(0.03)	0	(0)	1	(0.02)	0.58
Injury	Mi	5	(0.16)	0	(0)	5	(0.12)	0.22
Injury	Mo	1	(0.03)	0	(0)	1	(0.02)	0.58
Injury	S	1	(0.03)	0	(0)	1	(0.02)	0.58
Traumatic Injury	Mi	3	(0.09)	2	(0.21)	5	(0.12)	0.36
Traumatic Injury	Mo	2	(0.06)	0	(0)	2	(0.04)	0.44
Joint Dislocation	Mo	1	(0.03)	0	(0)	1	(0.02)	0.58
Postoperative Hypothyroidism	Mi	0	(0)	1	(0.1)	1	(0.02)	0.07
Animal Bite	Mi	1	(0.03)	0	(0)	1	(0.02)	0.58

SOC<br>PT	Severity	AVX	%	AZ	%	Total	%	p-Value
Arthropod Sting	Mi	1	(0.03)	0	(0)	1	(0.02)	0.58
Thermal Burn	Mo	1	(0.03)	0	(0)	1	(0.02)	0.58
Thermal Burn	S	1	(0.03)	0	(0)	1	(0.02)	0.58
Medical and Surgical Procedures	**Total**	22	(0.7)	2	(0.21)	24	(0.59)	0.08
	**Mi**	15	(0.48)	2	(0.21)	17	(0.41)	0.26
	**Mo**	7	(0.22)	0	(0)	7	(0.17)	0.14
	**S**	0	(0)	0	(0)	0	(0)	
Dental Care	Mi	1	(0.03)	0	(0)	1	(0.02)	0.58
Cataract Surgery	Mo	1	(0.03)	0	(0)	1	(0.02)	0.58
Nasal Surgery	Mi	1	(0.03)	0	(0)	1	(0.02)	0.58
Endodontics	Mi	1	(0.03)	0	(0)	1	(0.02)	0.58
Dental Extraction	Mi	5	(0.16)	2	(0.21)	7	(0.17)	0.72
Dental Extraction	Mo	5	(0.16)	0	(0)	5	(0.12)	0.22
Liposuction Removal	Mi	1	(0.03)	0	(0)	1	(0.02)	0.58
Liposuction Removal	Mo	1	(0.03)	0	(0)	1	(0.02)	0.58
Contraceptive Implant	Mi	1	(0.03)	0	(0)	1	(0.02)	0.58
Dental Implant	Mi	1	(0.03)	0	(0)	1	(0.02)	0.58
Transurethral Prostate Incision	Mi	1	(0.03)	0	(0)	1	(0.02)	0.58
Tooth Restoration	Mi	1	(0.03)	0	(0)	1	(0.02)	0.58
Rhinoplasty	Mi	2	(0.06)	0	(0)	2	(0.04)	0.44
Vasectomy	Mi	1	(0.03)	0	(0)	1	(0.02)	0.58
Cardiac Disorders	**Total**	12	(0.38)	5	(0.53)	17	(0.41)	0.53
	**Mi**	9	(0.28)	4	(0.42)	13	(0.32)	0.50
	**Mo**	3	(0.09)	0	(0)	3	(0.07)	0.34
	**S**	0	(0)	1	(0.1)	1	(0.02)	0.07
Arrhythmia	Mi	0	(0)	1	(0.1)	1	(0.02)	0.07
Chest Pain	Mi	2	(0.06)	1	(0.1)	3	(0.07)	0.67

SOC PT	Severity	AVX	%	AZ	%	Total	%	p-Value
Peripheral Edema	Mi	2	(0.06)	0	(0)	2	(0.04)	0.44
Peripheral Edema	Mo	2	(0.06)	0	(0)	2	(0.04)	0.44
Myocardial Infarction	S	0	(0)	1	(0.1)	1	(0.02)	0.07
Palpitations	Mi	3	(0.09)	1	(0.1)	4	(0.09)	0.92
Tachycardia	Mi	2	(0.06)	1	(0.1)	3	(0.07)	0.67
Tachycardia	Mo	1	(0.03)	0	(0)	1	(0.02)	0.58
Skin and Subcutaneous Tissue Disorders	**Total**	61	(1.95)	12	(1.28)	73	(1.79)	0.17
	**Mi**	45	(1.44)	12	(1.28)	57	(1.4)	0.71
	**Mo**	15	(0.48)	1	(0.1)	16	(0.39)	0.11
	**S**	3	(0.09)	0	(0)	3	(0.07)	0.34
Acne	Mi	2	(0.06)	1	(0.1)	3	(0.07)	0.67
Acne	Mo	1	(0.03)	0	(0)	1	(0.02)	0.58
Alopecia	Mi	5	(0.16)	1	(0.1)	6	(0.14)	0.71
Dermatitis	Mi	2	(0.06)	1	(0.1)	3	(0.07)	0.67
Dermatitis	Mo	3	(0.09)	0	(0)	3	(0.07)	0.34
Dermatitis	S	1	(0.03)	0	(0)	1	(0.02)	0.58
Ecchymosis	Mi	1	(0.03)	0	(0)	1	(0.02)	0.58
Erythema	Mi	0	(0)	1	(0.1)	1	(0.02)	0.07
Erythema	Mo	1	(0.03)	0	(0)	1	(0.02)	0.58
Palmar Erythema	Mi	1	(0.03)	0	(0)	1	(0.02)	0.58
Rash	Mi	2	(0.06)	0	(0)	2	(0.04)	0.44
Rash	Mo	1	(0.03)	0	(0)	1	(0.02)	0.58
Erythematous Rash	Mi	2	(0.06)	0	(0)	2	(0.04)	0.44
Skin Exfoliation	Mi	1	(0.03)	0	(0)	1	(0.02)	0.58
Hyperhidrosis	Mi	1	(0.03)	2	(0.21)	3	(0.07)	0.07
Hyperhidrosis	S	1	(0.03)	0	(0)	1	(0.02)	0.58
Skin Hypopigmentation	Mi	0	(0)	1	(0.1)	1	(0.02)	0.07

SOC PT	Severity	AVX	%	AZ	%	Total	%	p-Value
Skin Lesion	Mi	1	(0.03)	0	(0)	1	(0.02)	0.58
Skin Lesion	Mo	0	(0)	1	(0.1)	1	(0.02)	0.07
Lichen Sclerosus	Mi	1	(0.03)	0	(0)	1	(0.02)	0.58
Papule	Mi	3	(0.09)	1	(0.1)	4	(0.09)	0.92
Petechiae	Mi	1	(0.03)	0	(0)	1	(0.02)	0.58
Diabetic Foot	S	1	(0.03)	0	(0)	1	(0.02)	0.58
Pityriasis Rosea	Mi	1	(0.03)	0	(0)	1	(0.02)	0.58
Pruritus	Mi	16	(0.51)	4	(0.42)	20	(0.49)	0.74
Pruritus	Mo	7	(0.22)	0	(0)	7	(0.17)	0.14
Psoriasis	Mi	0	(0)	1	(0.1)	1	(0.02)	0.07
Rosacea	Mi	0	(0)	1	(0.1)	1	(0.02)	0.07
Urticaria	Mi	3	(0.09)	0	(0)	3	(0.07)	0.34
Urticaria	Mo	1	(0.03)	0	(0)	1	(0.02)	0.58
Skin Ulcer	Mo	1	(0.03)	0	(0)	1	(0.02)	0.58
Ingrown Nail	Mi	2	(0.06)	0	(0)	2	(0.04)	0.44
Vitiligo	Mi	1	(0.03)	0	(0)	1	(0.02)	0.58
Blood and Lymphatic System Disorders	**Total**	12	(0.38)	1	(0.1)	13	(0.32)	0.18
	**Mi**	8	(0.25)	0	(0)	8	(0.19)	0.12
	**Mo**	2	(0.06)	1	(0.1)	3	(0.07)	0.67
	**S**	2	(0.06)	0	(0)	2	(0.04)	0.44
Anemia	Mi	4	(0.12)	0	(0)	4	(0.09)	0.27
Anemia	Mo	1	(0.03)	0	(0)	1	(0.02)	0.58
Hypochromic Anemia	Mo	0	(0)	1	(0.1)	1	(0.02)	0.07
Pain in Lymph Node	Mi	1	(0.03)	0	(0)	1	(0.02)	0.58
Leukocytosis	Mi	1	(0.03)	0	(0)	1	(0.02)	0.58
Lymphadenitis	Mi	2	(0.06)	0	(0)	2	(0.04)	0.44
Lymphadenitis	Mo	1	(0.03)	0	(0)	1	(0.02)	0.58

SOC PT	Severity	AVX	%	AZ	%	Total	%	p-Value
Lymphadenitis	S	2	(0.06)	0	(0)	2	(0.04)	0.44
Reproductive System and Breast Disorders	**Total**	28	(0.89)	6	(0.64)	34	(0.83)	0.45
	**Mi**	22	(0.7)	5	(0.53)	27	(0.66)	0.57
	**Mo**	5	(0.16)	1	(0.1)	6	(0.14)	0.71
	**S**	1	(0.03)	0	(0)	1	(0.02)	0.58
Incomplete Abortion	S	1	(0.03)	0	(0)	1	(0.02)	0.58
Adenomyosis	Mi	1	(0.03)	0	(0)	1	(0.02)	0.58
Mammary Calcifications	Mi	0	(0)	1	(0.1)	1	(0.02)	0.07
Cystocele	Mi	1	(0.03)	0	(0)	1	(0.02)	0.58
Dysmenorrhea	Mi	6	(0.19)	1	(0.1)	7	(0.17)	0.58
Breast Pain	Mo	0	(0)	1	(0.1)	1	(0.02)	0.07
Penile Erythema	Mo	1	(0.03)	0	(0)	1	(0.02)	0.58
Vaginal Hemorrhage	Mi	1	(0.03)	0	(0)	1	(0.02)	0.58
Benign Prostatic Hyperplasia	Mi	2	(0.06)	0	(0)	2	(0.04)	0.44
Oligomenorrhea	Mi	1	(0.03)	0	(0)	1	(0.02)	0.58
Polycystic Ovaries	Mi	2	(0.06)	0	(0)	2	(0.04)	0.44
Polymenorrhea	Mi	0	(0)	1	(0.1)	1	(0.02)	0.07
Polymenorrhea	Mo	1	(0.03)	0	(0)	1	(0.02)	0.58
Breast Cyst	Mi	1	(0.03)	0	(0)	1	(0.02)	0.58
Breast Cyst	Mo	1	(0.03)	0	(0)	1	(0.02)	0.58
Ovarian Cyst	Mi	2	(0.06)	1	(0.1)	3	(0.07)	0.67
Uterine Cyst	Mi	0	(0)	1	(0.1)	1	(0.02)	0.07
Heavy Menstrual Bleeding	Mi	4	(0.12)	0	(0)	4	(0.09)	0.27
Heavy Menstrual Bleeding	Mo	1	(0.03)	0	(0)	1	(0.02)	0.58
Menopausal Symptoms	Mo	1	(0.03)	0	(0)	1	(0.02)	0.58
Menstrual Disorders	Mi	1	(0.03)	0	(0)	1	(0.02)	0.58
Metabolism and Nutrition Disorders	**Total**	108	(3.46)	20	(2.13)	128	(3.15)	0.04

SOC<br>PT	Severity	AVX	%	AZ	%	Total	%	p-Value
	**Mi**	99	(3.17)	19	(2.02)	118	(2.9)	0.06
	**Mo**	10	(0.32)	1	(0.1)	11	(0.27)	0.27
	**S**	0	(0)	0	(0)	0	(0)	
Glucose Tolerance Impairment	Mi	1	(0.03)	1	(0.1)	2	(0.04)	0.36
Decreased Appetite	Mi	0	(0)	1	(0.1)	1	(0.02)	0.07
Dehydration	Mi	1	(0.03)	1	(0.1)	2	(0.04)	0.36
Diabetes Mellitus	Mi	2	(0.06)	1	(0.1)	3	(0.07)	0.67
Dyslipidemia	Mi	5	(0.16)	1	(0.1)	6	(0.14)	0.71
Dyslipidemia	Mo	2	(0.06)	0	(0)	2	(0.04)	0.44
Vitamin D Deficiency	Mi	3	(0.09)	0	(0)	3	(0.07)	0.34
Hypercholesterolemia	Mi	5	(0.16)	0	(0)	5	(0.12)	0.22
Hypercholesterolemia	Mo	2	(0.06)	0	(0)	2	(0.04)	0.44
Hyperglycemia	Mi	1	(0.03)	1	(0.1)	2	(0.04)	0.36
Hypertriglyceridemia	Mi	2	(0.06)	2	(0.21)	4	(0.09)	0.20
Hypertriglyceridemia	Mo	2	(0.06)	0	(0)	2	(0.04)	0.44
Hyperuricemia	Mi	2	(0.06)	0	(0)	2	(0.04)	0.44
Hyperuricemia	Mo	1	(0.03)	1	(0.1)	2	(0.04)	0.36
Hypoglycemia	Mo	1	(0.03)	0	(0)	1	(0.02)	0.58
Lactose Intolerance	Mi	1	(0.03)	0	(0)	1	(0.02)	0.58
Obesity	Mi	34	(1.08)	7	(0.74)	41	(1.01)	0.35
Obesity	Mo	3	(0.09)	0	(0)	3	(0.07)	0.34
Polydipsia	Mi	4	(0.12)	0	(0)	4	(0.09)	0.27
Insulin Resistance	Mi	3	(0.09)	1	(0.1)	4	(0.09)	0.92
Overweight	Mi	46	(1.47)	5	(0.53)	51	(1.25)	0.02
Ear and Labyrinth Disorders	**Total**	19	(0.6)	4	(0.42)	23	(0.56)	0.51
	**Mi**	15	(0.48)	3	(0.32)	18	(0.44)	0.51
	**Mo**	4	(0.12)	1	(0.1)	5	(0.12)	0.87

SOC PT	Severity	AVX	%	AZ	%	Total	%	p-Value
	**S**	0	(0)	0	(0)	0	(0)	
Tinnitus	Mi	1	(0.03)	0	(0)	1	(0.02)	0.58
Ear Pain	Mi	5	(0.16)	1	(0.1)	6	(0.14)	0.71
Ear Pain	Mo	4	(0.12)	0	(0)	4	(0.09)	0.27
Hearing Loss	Mi	2	(0.06)	0	(0)	2	(0.04)	0.44
Ear Disorder	Mi	1	(0.03)	0	(0)	1	(0.02)	0.58
Outer Ear Disorder	Mi	1	(0.03)	0	(0)	1	(0.02)	0.58
Vertigo	Mi	5	(0.16)	2	(0.21)	7	(0.17)	0.72
Vertigo	Mo	0	(0)	1	(0.1)	1	(0.02)	0.07
Immune System Disorders	**Total**	7	(0.22)	0	(0)	7	(0.17)	0.14
	**Mi**	7	(0.22)	0	(0)	7	(0.17)	0.14
	**Mo**	0	(0)	0	(0)	0	(0)	
	**S**	0	(0)	0	(0)	0	(0)	
Hypersensitivity	Mi	7	(0.22)	0	(0)	7	(0.17)	0.14
Nervous System Disorders	**Total**	366	(11.73)	114	(12.17)	480	(11.83)	0.70
	**Mi**	273	(8.75)	91	(9.72)	364	(8.97)	0.36
	**Mo**	96	(3.07)	26	(2.77)	122	(3)	0.63
	**S**	14	(0.44)	3	(0.32)	17	(0.41)	0.59
Stroke	Mi	1	(0.03)	0	(0)	1	(0.02)	0.58
Stroke	S	1	(0.03)	0	(0)	1	(0.02)	0.58
Ageusia	Mi	1	(0.03)	1	(0.1)	2	(0.04)	0.36
Anosmia	Mi	0	(0)	1	(0.1)	1	(0.02)	0.07
Headache	Mi	231	(7.4)	81	(8.65)	312	(7.69)	0.20
Headache	Mo	86	(2.75)	25	(2.67)	111	(2.73)	0.88
Headache	S	10	(0.32)	1	(0.1)	11	(0.27)	0.27
Sciatica	Mi	2	(0.06)	0	(0)	2	(0.04)	0.44
Dysgeusia	Mi	0	(0)	1	(0.1)	1	(0.02)	0.07

SOC PT	Severity	AVX	%	AZ	%	Total	%	p-Value
Hyperesthesia	Mi	2	(0.06)	0	(0)	2	(0.04)	0.44
Hypoesthesia	Mi	1	(0.03)	1	(0.1)	2	(0.04)	0.36
Dizziness	Mi	18	(0.57)	8	(0.85)	26	(0.64)	0.35
Dizziness	Mo	3	(0.09)	0	(0)	3	(0.07)	0.34
Dizziness	S	0	(0)	1	(0.1)	1	(0.02)	0.07
Migraine	Mi	4	(0.12)	3	(0.32)	7	(0.17)	0.21
Migraine	Mo	3	(0.09)	0	(0)	3	(0.07)	0.34
Neuralgia	Mi	1	(0.03)	0	(0)	1	(0.02)	0.58
Diabetic Neuropathy	Mi	1	(0.03)	0	(0)	1	(0.02)	0.58
Diabetic Neuropathy	Mo	0	(0)	1	(0.1)	1	(0.02)	0.07
Peripheral Neuropathy	Mo	1	(0.03)	0	(0)	1	(0.02)	0.58
Brain Fog	Mi	1	(0.03)	0	(0)	1	(0.02)	0.58
Facial Paralysis	S	0	(0)	1	(0.1)	1	(0.02)	0.07
Paresthesia	Mi	10	(0.32)	1	(0.1)	11	(0.27)	0.27
Paresthesia	Mo	2	(0.06)	0	(0)	2	(0.04)	0.44
Loss of Muscle Strength	Mi	1	(0.03)	0	(0)	1	(0.02)	0.58
Drowsiness	Mi	12	(0.38)	1	(0.1)	13	(0.32)	0.18
Drowsiness	Mo	3	(0.09)	0	(0)	3	(0.07)	0.34
Drowsiness	S	4	(0.12)	1	(0.1)	5	(0.12)	0.87
Syncope	Mi	2	(0.06)	0	(0)	2	(0.04)	0.44
Carpal Tunnel Syndrome	Mi	1	(0.03)	0	(0)	1	(0.02)	0.58
Tremor	Mi	1	(0.03)	0	(0)	1	(0.02)	0.58
Endocrine Disorders	**Total**	3	(0.09)	2	(0.21)	5	(0.12)	0.36
	**Mi**	2	(0.06)	1	(0.1)	3	(0.07)	0.67
	**Mo**	1	(0.03)	1	(0.1)	2	(0.04)	0.36
	**S**	0	(0)	0	(0)	0	(0)	
Hyperthyroidism	Mo	0	(0)	1	(0.1)	1	(0.02)	0.07

SOC PT	Severity	AVX	%	AZ	%	Total	%	p-Value
Hypothyroidism	Mi	1	(0.03)	0	(0)	1	(0.02)	0.58
Hypothyroidism	Mo	1	(0.03)	0	(0)	1	(0.02)	0.58
Thyroid Mass	Mi	1	(0.03)	0	(0)	1	(0.02)	0.58
Thyroid Nodule	Mi	0	(0)	1	(0.1)	1	(0.02)	0.07
Gastrointestinal Disorders	**Total**	191	(6.12)	50	(5.34)	241	(5.94)	0.37
	**Mi**	147	(4.71)	47	(5.02)	194	(4.78)	0.69
	**Mo**	47	(1.5)	4	(0.42)	51	(1.25)	0.009
	**S**	3	(0.09)	1	(0.1)	4	(0.09)	0.92
Blisters on Oral Mucosa	Mi	1	(0.03)	0	(0)	1	(0.02)	0.58
Acute Abdomen	Mo	1	(0.03)	0	(0)	1	(0.02)	0.58
Dental caries	Mi	0	(0)	1	(0.1)	1	(0.02)	0.07
Dental caries	Mo	1	(0.03)	0	(0)	1	(0.02)	0.58
Colitis	Mi	5	(0.16)	0	(0)	5	(0.12)	0.22
Oral mucosa discoloration	Mi	0	(0)	1	(0.1)	1	(0.02)	0.07
Diarrhea	Mi	52	(1.66)	13	(1.38)	65	(1.6)	0.55
Diarrhea	Mo	21	(0.67)	1	(0.1)	22	(0.54)	0.03
Diarrhea	S	2	(0.06)	0	(0)	2	(0.04)	0.44
Abdominal distension	Mi	5	(0.16)	1	(0.1)	6	(0.14)	0.71
Abdominal distension	Mo	1	(0.03)	0	(0)	1	(0.02)	0.58
Abdominal pain	Mi	7	(0.22)	5	(0.53)	12	(0.29)	0.12
Abdominal pain	Mo	6	(0.19)	1	(0.1)	7	(0.17)	0.58
Abdominal pain	S	0	(0)	1	(0.1)	1	(0.02)	0.07
Dental pain	Mi	3	(0.09)	0	(0)	3	(0.07)	0.34
Upper abdominal pain	Mi	3	(0.09)	1	(0.1)	4	(0.09)	0.92
Gastroesophageal reflux disease	Mi	3	(0.09)	0	(0)	3	(0.07)	0.34
Constipation	Mi	3	(0.09)	1	(0.1)	4	(0.09)	0.92
Constipation	Mo	1	(0.03)	0	(0)	1	(0.02)	0.58

SOC PT	Severity	AVX	%	AZ	%	Total	%	p-Value
Gastritis	Mi	8	(0.25)	0	(0)	8	(0.19)	0.12
Gastritis	Mo	2	(0.06)	0	(0)	2	(0.04)	0.44
Glossitis	Mi	1	(0.03)	0	(0)	1	(0.02)	0.58
Glossodynia	Mi	1	(0.03)	0	(0)	1	(0.02)	0.58
Lumbar hernia	Mi	0	(0)	1	(0.1)	1	(0.02)	0.07
Food poisoning	Mi	1	(0.03)	0	(0)	1	(0.02)	0.58
Abdominal mass	Mi	1	(0.03)	0	(0)	1	(0.02)	0.58
Nausea	Mi	8	(0.25)	8	(0.85)	16	(0.39)	0.01
Nausea	Mo	2	(0.06)	1	(0.1)	3	(0.07)	0.67
Odynophagia	Mi	51	(1.63)	15	(1.6)	66	(1.62)	0.94
Odynophagia	Mo	10	(0.32)	1	(0.1)	11	(0.27)	0.27
Acute Pancreatitis	S	1	(0.03)	0	(0)	1	(0.02)	0.58
Irritable Bowel Syndrome	Mi	3	(0.09)	2	(0.21)	5	(0.12)	0.36
Irritable Bowel Syndrome	Mo	3	(0.09)	0	(0)	3	(0.07)	0.34
Vomiting	Mi	6	(0.19)	3	(0.32)	9	(0.22)	0.46
Vomiting	Mo	1	(0.03)	1	(0.1)	2	(0.04)	0.36
Aphthous Ulcer	Mi	2	(0.06)	1	(0.1)	3	(0.07)	0.67
Peptic Ulcer	Mi	1	(0.03)	1	(0.1)	2	(0.04)	0.36
General Disorders and Administration Site Reactions	**Total**	915	(29.32)	257	(27.45)	1172	(28.89)	0.26
	**Mi**	806	(25.83)	238	(25.42)	1044	(25.73)	0.80
	**Mo**	140	(4.48)	33	(3.52)	173	(4.26)	0.20
	**S**	16	(0.51)	1	(0.1)	17	(0.41)	0.09
Asthenia	Mi	23	(0.73)	5	(0.53)	28	(0.69)	0.51
Asthenia	Mo	4	(0.12)	0	(0)	4	(0.09)	0.27
Heat at the Vaccination Site	Mi	1	(0.03)	0	(0)	1	(0.02)	0.58
Pain at the Application Site	Mi	3	(0.09)	0	(0)	3	(0.07)	0.34
Pain at the Injection Site	Mi	657	(21.05)	166	(17.73)	823	(20.29)	0.02

SOC PT	Severity	AVX	%	AZ	%	Total	%	p-Value
Pain at the Injection Site	Mo	98	(3.14)	21	(2.24)	119	(2.93)	0.15
Pain at the Injection Site	S	8	(0.25)	0	(0)	8	(0.19)	0.12
Chest Pain	Mi	3	(0.09)	0	(0)	3	(0.07)	0.34
Edema at the Injection Site	Mi	4	(0.12)	1	(0.1)	5	(0.12)	0.87
Edema at the Injection Site	Mo	5	(0.16)	0	(0)	5	(0.12)	0.22
Flu-like Illness	Mi	16	(0.51)	5	(0.53)	21	(0.51)	0.93
Flu-like Illness	Mo	1	(0.03)	1	(0.1)	2	(0.04)	0.36
Influenza-like Illness	Mi	3	(0.09)	4	(0.42)	7	(0.17)	0.03
Erythema at the Injection Site	Mi	28	(0.89)	9	(0.96)	37	(0.91)	0.85
Erythema at the Injection Site	Mo	7	(0.22)	0	(0)	7	(0.17)	0.14
Erythema at the Injection Site	S	1	(0.03)	0	(0)	1	(0.02)	0.58
Chills	Mi	11	(0.35)	16	(1.7)	27	(0.66)	0.000
Chills	Mo	3	(0.09)	1	(0.1)	4	(0.09)	0.92
Chills	S	1	(0.03)	0	(0)	1	(0.02)	0.58
Fatigue	Mi	110	(3.52)	30	(3.2)	140	(3.45)	0.63
Fatigue	Mo	32	(1.02)	9	(0.96)	41	(1.01)	0.86
Fatigue	S	7	(0.22)	0	(0)	7	(0.17)	0.14
Peripheral Coldness	Mi	2	(0.06)	0	(0)	2	(0.04)	0.44
Inflammation	Mo	1	(0.03)	0	(0)	1	(0.02)	0.58
Hematoma at the Injection Site	Mi	1	(0.03)	1	(0.1)	2	(0.04)	0.36
Swelling	Mi	4	(0.12)	2	(0.21)	6	(0.14)	0.55
Hyperthermia	Mi	5	(0.16)	3	(0.32)	8	(0.19)	0.33
Induration at the Injection Site	Mi	11	(0.35)	3	(0.32)	14	(0.34)	0.88
Inflammation	Mi	2	(0.06)	1	(0.1)	3	(0.07)	0.67
Inflammation at the Injection Site	Mi	13	(0.41)	1	(0.1)	14	(0.34)	0.15
Nerve Injury at the Injection Site	Mi	0	(0)	2	(0.21)	2	(0.04)	0.009
Discomfort	Mi	2	(0.06)	0	(0)	2	(0.04)	0.44
Discomfort	Mo	2	(0.06)	0	(0)	2	(0.04)	0.44

SOC PT	Severity	AVX	%	AZ	%	Total	%	p-Value
Discomfort	S	1	(0.03)	0	(0)	1	(0.02)	0.58
General Malaise	Mi	24	(0.76)	9	(0.96)	33	(0.81)	0.56
General Malaise	Mo	4	(0.12)	1	(0.1)	5	(0.12)	0.87
Chest Tightness	Mi	2	(0.06)	1	(0.1)	3	(0.07)	0.67
Pyrexia	Mi	55	(1.76)	53	(5.66)	108	(2.66)	0.000
Pyrexia	Mo	19	(0.6)	4	(0.42)	23	(0.56)	0.51
Pyrexia	S	2	(0.06)	1	(0.1)	3	(0.07)	0.67
Pruritus at the Injection Site	Mi	0	(0)	1	(0.1)	1	(0.02)	0.07
Cold Sensation	Mi	0	(0)	1	(0.1)	1	(0.02)	0.07
Decreased Thirst	Mi	0	(0)	1	(0.1)	1	(0.02)	0.07
Chronic Fatigue Syndrome	Mi	1	(0.03)	0	(0)	1	(0.02)	0.58
Mucosal Disorder	Mi	1	(0.03)	0	(0)	1	(0.02)	0.58
Xerosis	Mi	1	(0.03)	0	(0)	1	(0.02)	0.58
Xerosis	Mo	1	(0.03)	0	(0)	1	(0.02)	0.58
Hepatobiliary Disorders	**Total**	6	(0.19)	2	(0.21)	8	(0.19)	0.89
	**Mi**	3	(0.09)	2	(0.21)	5	(0.12)	0.36
	**Mo**	2	(0.06)	0	(0)	2	(0.04)	0.44
	**S**	1	(0.03)	0	(0)	1	(0.02)	0.58
Acute Cholecystitis	Mo	1	(0.03)	0	(0)	1	(0.02)	0.58
Cholelithiasis	Mi	1	(0.03)	0	(0)	1	(0.02)	0.58
Cholelithiasis	Mo	1	(0.03)	0	(0)	1	(0.02)	0.58
Cholelithiasis	S	1	(0.03)	0	(0)	1	(0.02)	0.58
Biliary Secretion Deficiency	Mi	1	(0.03)	0	(0)	1	(0.02)	0.58
Hepatic Steatosis	Mi	1	(0.03)	1	(0.1)	2	(0.04)	0.36
Jaundice	Mi	0	(0)	1	(0.1)	1	(0.02)	0.07
Musculoskeletal and Connective Tissue Disorders	**Total**	221	(7.08)	71	(7.58)	292	(7.19)	0.60
	**Mi**	153	(4.9)	58	(6.19)	211	(5.2)	0.11

SOC<br>PT	Severity	AVX	%	AZ	%	Total	%	p-Value
	**Mo**	73	(2.33)	12	(1.28)	85	(2.09)	0.04
	**S**	5	(0.16)	1	(0.1)	6	(0.14)	0.71
Arthralgia	Mi	34	(1.08)	22	(2.35)	56	(1.38)	0.003
Arthralgia	Mo	17	(0.54)	4	(0.42)	21	(0.51)	0.66
Arthralgia	S	1	(0.03)	0	(0)	1	(0.02)	0.58
Bursitis	Mi	1	(0.03)	0	(0)	1	(0.02)	0.58
Contusion	Mo	4	(0.12)	0	(0)	4	(0.09)	0.27
Costochondritis	Mo	1	(0.03)	1	(0.1)	2	(0.04)	0.36
Neck Pain	Mi	1	(0.03)	2	(0.21)	3	(0.07)	0.07
Back Pain	Mi	17	(0.54)	6	(0.64)	23	(0.56)	0.73
Back Pain	Mo	12	(0.38)	1	(0.1)	13	(0.32)	0.18
Back Pain	S	1	(0.03)	0	(0)	1	(0.02)	0.58
Shoulder Pain	Mi	1	(0.03)	0	(0)	1	(0.02)	0.58
Pain in a Limb	Mi	10	(0.32)	0	(0)	10	(0.24)	0.08
Pain in a Limb	Mo	5	(0.16)	0	(0)	5	(0.12)	0.22
Ligament Sprain	Mi	4	(0.12)	3	(0.32)	7	(0.17)	0.21
Ligament Sprain	Mo	3	(0.09)	2	(0.21)	5	(0.12)	0.36
Muscle Spasms	Mi	4	(0.12)	3	(0.32)	7	(0.17)	0.21
Muscle Spasms	Mo	5	(0.16)	0	(0)	5	(0.12)	0.22
Exostosis	Mi	1	(0.03)	0	(0)	1	(0.02)	0.58
Plantar Fasciitis	Mo	1	(0.03)	0	(0)	1	(0.02)	0.58
Mass in Neck	Mo	1	(0.03)	0	(0)	1	(0.02)	0.58
Myalgia	Mi	77	(2.46)	26	(2.77)	103	(2.53)	0.59
Myalgia	Mo	34	(1.08)	5	(0.53)	39	(0.96)	0.12
Myalgia	S	3	(0.09)	1	(0.1)	4	(0.09)	0.92
Discomfort in Limbs	Mi	3	(0.09)	0	(0)	3	(0.07)	0.34
Discomfort in Limbs	Mo	1	(0.03)	1	(0.1)	2	(0.04)	0.36
Osteoarthritis	Mi	1	(0.03)	1	(0.1)	2	(0.04)	0.36

SOC PT	Severity	AVX	%	AZ	%	Total	%	p-Value
Osteopenia	Mi	2	(0.06)	0	(0)	2	(0.04)	0.44
Synovial Cyst	Mi	0	(0)	1	(0.1)	1	(0.02)	0.07
Synovitis	Mo	1	(0.03)	0	(0)	1	(0.02)	0.58
Tendonitis	Mi	1	(0.03)	0	(0)	1	(0.02)	0.58
Eye Disorders	**Total**	31	(0.99)	8	(0.85)	39	(0.96)	0.70
	**Mi**	20	(0.64)	7	(0.74)	27	(0.66)	0.72
	**Mo**	11	(0.35)	1	(0.1)	12	(0.29)	0.22
	**S**	0	(0)	0	(0)	0	(0)	
Visual disturbance	Mi	0	(0)	1	(0.1)	1	(0.02)	0.07
Blepharitis	Mo	0	(0)	1	(0.1)	1	(0.02)	0.07
Cataract	Mo	1	(0.03)	0	(0)	1	(0.02)	0.58
Chalazion	Mi	1	(0.03)	0	(0)	1	(0.02)	0.58
Conjunctivitis	Mi	8	(0.25)	1	(0.1)	9	(0.22)	0.39
Conjunctivitis	Mo	4	(0.12)	0	(0)	4	(0.09)	0.27
Allergic conjunctivitis	Mi	4	(0.12)	0	(0)	4	(0.09)	0.27
Allergic conjunctivitis	Mo	4	(0.12)	0	(0)	4	(0.09)	0.27
Palpebral edema	Mi	0	(0)	1	(0.1)	1	(0.02)	0.07
Photopsia	Mi	0	(0)	1	(0.1)	1	(0.02)	0.07
Eyelid furuncle	Mi	0	(0)	1	(0.1)	1	(0.02)	0.07
Conjunctival hemorrhage	Mi	1	(0.03)	1	(0.1)	2	(0.04)	0.36
Hemorrhage in the eye	Mi	1	(0.03)	0	(0)	1	(0.02)	0.58
Hemorrhage in the eye	Mo	1	(0.03)	0	(0)	1	(0.02)	0.58
Ocular hyperemia	Mi	1	(0.03)	0	(0)	1	(0.02)	0.58
Ocular hyperemia	Mo	1	(0.03)	0	(0)	1	(0.02)	0.58
Increased tearing	Mi	2	(0.06)	0	(0)	2	(0.04)	0.44
Itching in the eye	Mi	1	(0.03)	0	(0)	1	(0.02)	0.58
Abnormal sensation in the eye	Mi	0	(0)	1	(0.1)	1	(0.02)	0.07

SOC<br>PT	Severity	AVX	%	AZ	%	Total	%	p-Value
Blurred vision	Mi	1	(0.03)	0	(0)	1	(0.02)	0.58
Psychiatric disorders	**Total**	27	(0.86)	7	(0.74)	34	(0.83)	0.72
	**Mi**	19	(0.6)	7	(0.74)	26	(0.64)	0.64
	**Mo**	7	(0.22)	0	(0)	7	(0.17)	0.14
	**S**	1	(0.03)	0	(0)	1	(0.02)	0.58
Agitation	Mi	0	(0)	1	(0.1)	1	(0.02)	0.07
Anxiety	Mi	5	(0.16)	3	(0.32)	8	(0.19)	0.33
Anxiety	Mo	2	(0.06)	0	(0)	2	(0.04)	0.44
Mood changes	Mi	1	(0.03)	0	(0)	1	(0.02)	0.58
Depression	Mi	4	(0.12)	2	(0.21)	6	(0.14)	0.55
Depression	Mo	6	(0.19)	0	(0)	6	(0.14)	0.22
Depression	S	1	(0.03)	0	(0)	1	(0.02)	0.58
Disorientation	Mi	1	(0.03)	0	(0)	1	(0.02)	0.58
Confusional state	Mi	1	(0.03)	1	(0.1)	2	(0.04)	0.36
Insomnia	Mi	3	(0.09)	2	(0.21)	5	(0.12)	0.36
Irritability	Mi	2	(0.06)	1	(0.1)	3	(0.07)	0.67
Increased libido	Mi	0	(0)	1	(0.1)	1	(0.02)	0.07
Panic reaction	Mi	1	(0.03)	0	(0)	1	(0.02)	0.58
Post-traumatic stress disorder	Mo	1	(0.03)	0	(0)	1	(0.02)	0.58
Dissociative disorder	Mi	1	(0.03)	0	(0)	1	(0.02)	0.58
Attention deficit hyperactivity disorder	Mi	2	(0.06)	0	(0)	2	(0.04)	0.44
Renal and urinary disorders	**Total**	6	(0.19)	2	(0.21)	8	(0.19)	0.89
	**Mi**	3	(0.09)	1	(0.1)	4	(0.09)	0.92
	**Mo**	3	(0.09)	1	(0.1)	4	(0.09)	0.92
	**S**	0	(0)	0	(0)	0	(0)	
Chromaturia	Mo	1	(0.03)	0	(0)	1	(0.02)	0.58
Dysuria	Mi	1	(0.03)	0	(0)	1	(0.02)	0.58

SOC PT	Severity	AVX	%	AZ	%	Total	%	p-Value
Dysuria	Mo	1	(0.03)	0	(0)	1	(0.02)	0.58
Renal lithiasis	Mi	1	(0.03)	1	(0.1)	2	(0.04)	0.36
Renal lithiasis	Mo	0	(0)	1	(0.1)	1	(0.02)	0.07
Pyelocaliectasis	Mi	1	(0.03)	0	(0)	1	(0.02)	0.58
Polyuria	Mo	1	(0.03)	0	(0)	1	(0.02)	0.58
Respiratory, Thoracic and Mediastinal disorders	**Total**	356	(11.41)	102	(10.89)	458	(11.29)	0.66
	**Mi**	297	(9.51)	95	(10.14)	392	(9.66)	0.56
	**Mo**	70	(2.24)	9	(0.96)	79	(1.94)	0.01
	**S**	8	(0.25)	2	(0.21)	10	(0.24)	0.81
Aphonia	Mi	2	(0.06)	2	(0.21)	4	(0.09)	0.20
Tonsillitis	Mi	0	(0)	1	(0.1)	1	(0.02)	0.07
Asthma	Mi	1	(0.03)	2	(0.21)	3	(0.07)	0.07
Asthma	Mo	0	(0)	1	(0.1)	1	(0.02)	0.07
Increased secretion of the upper respiratory tract	Mi	1	(0.03)	0	(0)	1	(0.02)	0.58
Bronchospasm	Mo	1	(0.03)	0	(0)	1	(0.02)	0.58
Allergic bronchitis	Mo	1	(0.03)	0	(0)	1	(0.02)	0.58
Nasal congestion	Mi	18	(0.57)	3	(0.32)	21	(0.51)	0.33
Nasal congestion	Mo	3	(0.09)	0	(0)	3	(0.07)	0.34
Nasal congestion	S	1	(0.03)	0	(0)	1	(0.02)	0.58
Dysphonia	Mi	5	(0.16)	1	(0.1)	6	(0.14)	0.71
Dyspnea	Mi	5	(0.16)	1	(0.1)	6	(0.14)	0.71
Dyspnea	Mo	1	(0.03)	1	(0.1)	2	(0.04)	0.36
Dyspnea	S	1	(0.03)	0	(0)	1	(0.02)	0.58
Pharyngeal-laryngeal pain	Mi	23	(0.73)	11	(1.17)	34	(0.83)	0.19
Pharyngeal-laryngeal pain	Mo	12	(0.38)	1	(0.1)	13	(0.32)	0.18
Pharyngeal-laryngeal pain	S	2	(0.06)	1	(0.1)	3	(0.07)	0.67
Oropharyngeal pain	Mi	3	(0.09)	0	(0)	3	(0.07)	0.34
Chest pain	S	0	(0)	1	(0.1)	1	(0.02)	0.07

SOC PT	Severity	AVX	%	AZ	%	Total	%	p-Value
Pleuritic pain	Mi	2	(0.06)	0	(0)	2	(0.04)	0.44
Pharyngeal erythema	Mi	1	(0.03)	1	(0.1)	2	(0.04)	0.36
Epistaxis	Mi	1	(0.03)	0	(0)	1	(0.02)	0.58
Epistaxis	Mo	1	(0.03)	0	(0)	1	(0.02)	0.58
Rales	Mi	1	(0.03)	0	(0)	1	(0.02)	0.58
Sneezing	Mi	6	(0.19)	0	(0)	6	(0.14)	0.17
Sneezing	Mo	4	(0.12)	0	(0)	4	(0.09)	0.27
Sneezing	S	3	(0.09)	0	(0)	3	(0.07)	0.34
Pharyngotonsillitis	Mi	75	(2.4)	33	(3.52)	108	(2.66)	0.06
Pharyngotonsillitis	Mo	26	(0.83)	3	(0.32)	29	(0.71)	0.10
Pharyngotonsillitis	S	0	(0)	1	(0.1)	1	(0.02)	0.07
Dry throat	Mi	1	(0.03)	2	(0.21)	3	(0.07)	0.07
Dry throat	Mo	1	(0.03)	0	(0)	1	(0.02)	0.58
Hiccup	Mi	1	(0.03)	0	(0)	1	(0.02)	0.58
Throat irritation	Mi	6	(0.19)	0	(0)	6	(0.14)	0.34
Nasopharyngitis	Mi	32	(1.02)	8	(0.85)	40	(0.98)	0.64
Nasopharyngitis	Mo	0	(0)	1	(0.1)	1	(0.02)	0.07
Nasal itching	Mi	1	(0.03)	0	(0)	1	(0.02)	0.58
Allergic rhinitis	Mi	20	(0.64)	5	(0.53)	25	(0.61)	0.71
Allergic rhinitis	Mo	2	(0.06)	0	(0)	2	(0.04)	0.44
Rhinorrhea	Mi	92	(2.94)	28	(2.99)	120	(2.95)	0.94
Rhinorrhea	Mo	25	(0.8)	2	(0.21)	27	(0.66)	0.05
Rhinorrhea	S	5	(0.16)	0	(0)	5	(0.12)	0.22
Sleep apnea syndrome	Mi	0	(0)	1	(0.1)	1	(0.02)	0.07
Respiratory symptom	Mi	1	(0.03)	0	(0)	1	(0.02)	0.58
Cough	Mi	49	(1.57)	22	(2.35)	71	(1.75)	0.11
Cough	Mo	9	(0.28)	1	(0.1)	10	(0.24)	0.32

SOC PT	Severity	AVX	%	AZ	%	Total	%	p-Value
Cough	S	3	(0.09)	1	(0.1)	4	(0.09)	0.92
Productive cough	Mi	1	(0.03)	0	(0)	1	(0.02)	0.58
Vascular disorders	**Total**	46	(1.47)	13	(1.38)	59	(1.45)	0.84
	**Mi**	37	(1.18)	13	(1.38)	50	(1.23)	0.62
	**Mo**	8	(0.25)	1	(0.1)	9	(0.22)	0.39
	**S**	1	(0.03)	0	(0)	1	(0.02)	0.58
Hot flashes	Mi	3	(0.09)	1	(0.1)	4	(0.09)	0.92
Hypertensive crisis	Mo	1	(0.03)	0	(0)	1	(0.02)	0.58
Peripheral vascular disease	Mi	1	(0.03)	0	(0)	1	(0.02)	0.58
Hemorrhoids	Mo	1	(0.03)	0	(0)	1	(0.02)	0.58
Hypertension	Mi	3	(0.09)	1	(0.1)	4	(0.09)	0.92
Hypotension	Mo	2	(0.06)	0	(0)	2	(0.04)	0.44
Hot flashes	Mo	1	(0.03)	0	(0)	1	(0.02)	0.58
Hypertensive crisis	Mi	25	(0.8)	11	(1.17)	36	(0.88)	0.28
Peripheral vascular disease	Mo	2	(0.06)	0	(0)	2	(0.04)	0.44
Hemorrhoids	Mi	2	(0.06)	0	(0)	2	(0.04)	0.44
Hypotension	Mo	1	(0.03)	0	(0)	1	(0.02)	0.58
Lymphedema	Mi	1	(0.03)	0	(0)	1	(0.02)	0.58
Peripheral venous thrombosis	Mi	1	(0.03)	0	(0)	1	(0.02)	0.58
Hypertensive urgency	Mo	0	(0)	1	(0.1)	1	(0.02)	0.07
Varicose vein	S	1	(0.03)	0	(0)	1	(0.02)	0.58
Hypotension	Mi	1	(0.03)	0	(0)	1	(0.02)	0.58

SOC: System Organ Class; PT: Preferred term; AE: Adverse Event; Mi: Mild; Mo: Moderate; S: Severe; AVX: AVX/COVID-12; AZ: AZ/ChAdOx-1-S. P-value calculated from comparison of proportions (Z-test).

Table S4. Proportion of affected subjects by local adverse events of special interest (AESIs) at 7 days post-immunization

SOC<br>PT	Severity	AVX	%	AZ	%	Total	%	p-Value
Subjects		3120		936		4056		
Any AE	Total	800	(25.64)	193	(20.61)	993	(24.48)	0.001
	**Mi**	698	(22.37)	173	(18.48)	871	(21.47)	0.01
	**Mo**	108	(3.46)	21	(2.24)	129	(3.18)	0.06
	**S**	9	(0.28)	0	(0)	9	(0.22)	0.10
Skin and subcutaneous tissue disorders	**Total**	23	(0.73)	5	(0.53)	28	(0.69)	0.51
	**Mi**	17	(0.54)	5	(0.53)	22	(0.54)	0.96
	**Mo**	6	(0.19)	0	(0)	6	(0.14)	0.17
	**S**	0	(0)	0	(0)	0	(0)	
Erythema	Mo	1	(0.03)	0	(0)	1	(0.02)	0.58
Papule	Mi	2	(0.06)	1	(0.1)	3	(0.07)	0.67
Itching	Mi	15	(0.48)	4	(0.42)	19	(0.46)	0.83
Itching	Mo	5	(0.16)	0	(0)	5	(0.12)	0.22
Nervous system disorders	**Total**	6	(0.19)	0	(0)	6	(0.14)	0.17
	**Mi**	5	(0.16)	0	(0)	5	(0.12)	0.22
	**Mo**	1	(0.03)	0	(0)	1	(0.02)	0.58
	**S**	0	(0)	0	(0)	0	(0)	
Paresthesia	Mi	5	(0.16)	0	(0)	5	(0.12)	0.22
Paresthesia	Mo	1	(0.03)	0	(0)	1	(0.02)	0.58
General disorders and local events	**Total**	786	(25.19)	190	(20.29)	976	(24.06)	0.002
	**Mi**	685	(21.95)	170	(18.16)	855	(21.07)	0.01
	**Mo**	101	(3.23)	21	(2.24)	122	(3)	0.11
	**S**	9	(0.28)	0	(0)	9	(0.22)	0.10
Heat at the vaccination site	Mi	1	(0.03)	0	(0)	1	(0.02)	0.58
Pain at the application site	Mi	3	(0.09)	0	(0)	3	(0.07)	0.34
Pain at the application site	Mi	655	(20.99)	165	(17.62)	820	(20.21)	0.02

SOC PT	Severity	AVX	%	AZ	%	Total	%	p-Value
Pain at the injection site	Mo	98	(3.14)	21	(2.24)	119	(2.93)	0.15
Pain at the injection site	S	8	(0.25)	0	(0)	8	(0.19)	0.12
Edema at the injection site	Mi	4	(0.12)	1	(0.1)	5	(0.12)	0.87
Edema at the injection site	Mo	5	(0.16)	0	(0)	5	(0.12)	0.22
Redness at the injection site	Mi	28	(0.89)	9	(0.96)	37	(0.91)	0.85
Redness at the injection site	Mo	7	(0.22)	0	(0)	7	(0.17)	0.14
Redness at the injection site	S	1	(0.03)	0	(0)	1	(0.02)	0.58
Hematoma at the injection site	Mi	1	(0.03)	1	(0.1)	2	(0.04)	0.36
Swelling	Mi	4	(0.12)	2	(0.21)	6	(0.14)	0.55
Induration at the application site	Mi	11	(0.35)	3	(0.32)	14	(0.34)	0.88
Inflammation	Mi	13	(0.41)	1	(0.1)	14	(0.34)	0.15
Nerve injury at the injection site	Mi	0	(0)	2	(0.21)	2	(0.04)	0.009
Itching at the injection site	Mi	0	(0)	1	(0.1)	1	(0.02)	0.07
Musculoskeletal and connective tissue disorders	**Total**	10	(0.32)	0	(0)	10	(0.24)	0.08
	**Mi**	6	(0.19)	0	(0)	6	(0.14)	0.17
	**Mo**	4	(0.12)	0	(0)	4	(0.09)	0.27
	**S**	0	(0)	0	(0)	0	(0)	
Pain in a limb	Mi	4	(0.12)	0	(0)	4	(0.09)	0.27
Pain in a limb	Mo	3	(0.09)	0	(0)	3	(0.07)	0.34
Discomfort in the extremities	Mi	2	(0.06)	0	(0)	2	(0.04)	0.43
Discomfort in the extremities	Mo	1	(0.03)	0	(0)	1	(0.02)	0.58

SOC: System Organ Class; PT: Preferred term; AE: Adverse Event; Mi: Mild; Mo: Moderate; S: Severe; AVX: AVX/COVID-12; AZ: AZ/ChAdOx-1-S. P-value calculated from comparison of proportions (Z-test).

Table S5. Proportion of affected subjects by systemic adverse events of special interest (AESIs) at 7 days post-immunization

SOC<br>PT	Severity	AVX	%	AZ	%	Total	%	p-Value
Subjects		3120		936		4056		
Any AE	Total	596	(19.1)	203	(21.68)	799	(19.69)	0.08
	**Mi**	496	(15.89)	183	(19.55)	679	(16.74)	0.008
	**Mo**	139	(4.45)	39	(4.16)	178	(4.38)	0.70
	**S**	16	(0.51)	2	(0.21)	18	(0.44)	0.22
Supplementary examinations	**Total**	1	(0.03)	1	(0.1)	2	(0.04)	0.36
	**Mi**	1	(0.03)	1	(0.1)	2	(0.04)	0.36
	**Mo**	1	(0.03)	0	(0)	1	(0.02)	0.58
	**S**	0	(0)	0	(0)	0	(0)	
High blood pressure elevation	Mi	1	(0.03)	1	(0.1)	2	(0.04)	0.36
Infections and infestations	**Total**	2	(0.06)	0	(0)	2	(0.04)	0.43
	**Mi**	1	(0.03)	0	(0)	1	(0.02)	0.58
	**Mo**	1	(0.03)	0	(0)	1	(0.02)	0.58
	**S**	0	(0)	0	(0)	0	(0)	
Oral herpes	Mi	1	(0.03)	0	(0)	1	(0.02)	0.58
Oral herpes	Mo	1	(0.03)	0	(0)	1	(0.02)	0.58
Cardiac disorders	**Total**	3	(0.09)	3	(0.32)	6	(0.14)	0.11
	**Mi**	2	(0.06)	3	(0.32)	5	(0.12)	0.05
	**Mo**	1	(0.03)	0	(0)	1	(0.02)	0.58
	**S**	0	(0)	0	(0)	0	(0)	
Chest pain	Mi	0	(0)	1	(0.1)	1	(0.02)	0.07
Palpitations	Mi	1	(0.03)	1	(0.1)	2	(0.04)	0.36
Tachycardia	Mi	1	(0.03)	1	(0.1)	2	(0.04)	0.36
Tachycardia	Mo	1	(0.03)	0	(0)	1	(0.02)	0.58
Skin and subcutaneous tissue disorders	**Total**	6	(0.19)	2	(0.21)	8	(0.19)	0.89
	**Mi**	4	(0.12)	1	(0.1)	5	(0.12)	0.87

SOC<br>PT	Severity	AVX	%	AZ	%	Total	%	p-Value
	**Mo**	1	(0.03)	1	(0.1)	2	(0.04)	0.36
	**S**	1	(0.03)	0	(0)	1	(0.02)	0.58
Rash	Mi	2	(0.06)	0	(0)	2	(0.04)	0.43
Excessive sweating	Mi	0	(0)	1	(0.1)	1	(0.02)	0.07
Excessive sweating	S	1	(0.03)	0	(0)	1	(0.02)	0.58
Skin lesion	Mo	0	(0)	1	(0.1)	1	(0.02)	0.07
Hives	Mi	2	(0.06)	0	(0)	2	(0.04)	0.43
Hives	Mo	1	(0.03)	0	(0)	1	(0.02)	0.58
Reproductive and breast disorders	**Total**	3	(0.09)	1	(0.1)	4	(0.09)	0.92
	**Mi**	2	(0.06)	1	(0.1)	3	(0.07)	0.67
	**Mo**	1	(0.03)	0	(0)	1	(0.02)	0.58
	**S**	0	(0)	0	(0)	0	(0)	
Polymenorrhea	Mi	0	(0)	1	(0.1)	1	(0.02)	0.07
Polymenorrhea	Mo	1	(0.03)	0	(0)	1	(0.02)	0.58
Heavy menstrual bleeding	Mi	1	(0.03)	0	(0)	1	(0.02)	0.58
Menstrual disorders	Mi	1	(0.03)	0	(0)	1	(0.02)	0.58
Metabolism and nutrition disorders	**Total**	0	(0)	1	(0.1)	1	(0.02)	0.07
	**Mi**	0	(0)	1	(0.1)	1	(0.02)	0.07
	**Mo**	0	(0)	0	(0)	0	(0)	
	**S**	0	(0)	0	(0)	0	(0)	
Decreased appetite	Mi	0	(0)	1	(0.1)	1	(0.02)	0.07
Ear and labyrinth disorders	**Total**	2	(0.06)	1	(0.1)	3	(0.07)	0.67
	**Mi**	2	(0.06)	0	(0)	2	(0.04)	0.43
	**Mo**	0	(0)	1	(0.1)	1	(0.02)	0.07
	**S**	0	(0)	0	(0)	0	(0)	
Vertigo	Mi	2	(0.06)	0	(0)	2	(0.04)	0.43
Vertigo	Mo	0	(0)	1	(0.1)	1	(0.02)	0.07

SOC PT	Severity	AVX	%	AZ	%	Total	%	p-Value
Immune system disorders	**Total**	2	(0.06)	0	(0)	2	(0.04)	0.43
	**Mi**	2	(0.06)	0	(0)	2	(0.04)	0.43
	**Mo**	0	(0)	0	(0)	0	(0)	
	**S**	0	(0)	0	(0)	0	(0)	
Hypersensitivity	Mi	2	(0.06)	0	(0)	2	(0.04)	0.43
Nervous system disorders	**Total**	270	(8.65)	98	(10.47)	368	(9.07)	0.08
	**Mi**	193	(6.18)	77	(8.22)	270	(6.65)	0.02
	**Mo**	69	(2.21)	21	(2.24)	90	(2.21)	0.95
	**S**	10	(0.32)	1	(0.1)	11	(0.27)	0.27
Headache	Mi	171	(5.48)	72	(7.69)	243	(5.99)	0.01
Headache	Mo	66	(2.11)	21	(2.24)	87	(2.14)	0.81
Headache	S	8	(0.25)	0	(0)	8	(0.19)	0.12
Dizziness	Mi	15	(0.48)	7	(0.74)	22	(0.54)	0.32
Dizziness	Mo	2	(0.06)	0	(0)	2	(0.04)	0.43
Migraine	Mi	1	(0.03)	0	(0)	1	(0.02)	0.58
Paresthesia	Mi	2	(0.06)	0	(0)	2	(0.04)	0.43
Drowsiness	Mi	11	(0.35)	1	(0.1)	12	(0.29)	0.22
Drowsiness	Mo	3	(0.09)	0	(0)	3	(0.07)	0.34
Drowsiness	S	4	(0.12)	1	(0.1)	5	(0.12)	0.87
Tremor	Mi	1	(0.03)	0	(0)	1	(0.02)	0.58
Gastrointestinal disorders	**Total**	62	(1.98)	21	(2.24)	83	(2.04)	0.62
	**Mi**	47	(1.5)	19	(2.02)	66	(1.62)	0.26
	**Mo**	15	(0.48)	2	(0.21)	17	(0.41)	0.26
	**S**	1	(0.03)	1	(0.1)	2	(0.04)	0.36
Diarrhea	Mi	25	(0.8)	10	(1.06)	35	(0.86)	0.43
Diarrhea	Mo	8	(0.25)	1	(0.1)	9	(0.22)	0.39
Diarrhea	S	1	(0.03)	0	(0)	1	(0.02)	0.58

SOC	PT	Severity	AVX	%	AZ	%	Total	%	p-Value
	Abdominal distension	Mi	1	(0.03)	0	(0)	1	(0.02)	0.58
	Abdominal pain	Mi	5	(0.16)	1	(0.1)	6	(0.14)	0.70
	Abdominal pain	Mo	2	(0.06)	0	(0)	2	(0.04)	0.43
	Abdominal pain	S	0	(0)	1	(0.1)	1	(0.02)	0.07
	Upper abdominal pain	Mi	1	(0.03)	0	(0)	1	(0.02)	0.58
	Constipation	Mi	1	(0.03)	0	(0)	1	(0.02)	0.58
	Nausea	Mi	4	(0.12)	6	(0.64)	10	(0.24)	0.005
	Nausea	Mo	1	(0.03)	1	(0.1)	2	(0.04)	0.36
	Odynophagia	Mi	12	(0.38)	4	(0.42)	16	(0.39)	0.85
	Odynophagia	Mo	4	(0.12)	0	(0)	4	(0.09)	0.27
	Vomiting	Mi	2	(0.06)	1	(0.1)	3	(0.07)	0.67
	Vomiting	Mo	0	(0)	1	(0.1)	1	(0.02)	0.07
	Aphthous ulcer	Mi	1	(0.03)	0	(0)	1	(0.02)	0.58
General disorders and administration site alterations		**Total**	255	(8.17)	110	(11.75)	365	(8.99)	0.0008
		**Mi**	204	(6.53)	99	(10.57)	303	(7.47)	0.000
		**Mo**	50	(1.6)	14	(1.49)	64	(1.57)	0.81
		**S**	7	(0.22)	0	(0)	7	(0.17)	0.14
	Asthenia	Mi	21	(0.67)	3	(0.32)	24	(0.59)	0.21
	Asthenia	Mo	2	(0.06)	0	(0)	2	(0.04)	0.43
	Chest pain	Mi	1	(0.03)	0	(0)	1	(0.02)	0.58
	Flu-like illness	Mi	2	(0.06)	0	(0)	2	(0.04)	0.43
	Flu-like illness	Mo	1	(0.03)	1	(0.1)	2	(0.04)	0.36
	Influenza-like illness	Mi	0	(0)	1	(0.1)	1	(0.02)	0.07
	Shivering	Mi	8	(0.25)	16	(1.7)	24	(0.59)	0.000
	Shivering	Mo	3	(0.09)	1	(0.1)	4	(0.09)	0.92
	Shivering	S	1	(0.03)	0	(0)	1	(0.02)	0.58
	Fatigue	Mi	109	(3.49)	29	(3.09)	138	(3.4)	0.55
	Fatigue	Mo	30	(0.96)	9	(0.96)	39	(0.96)	0.99

SOC PT	Severity	AVX	%	AZ	%	Total	%	p-Value
Fatigue	S	6	(0.19)	0	(0)	6	(0.14)	0.17
Peripheral coldness	Mi	2	(0.06)	0	(0)	2	(0.04)	0.43
Hyperthermia	Mi	5	(0.16)	3	(0.32)	8	(0.19)	0.33
Inflammation	Mi	1	(0.03)	0	(0)	1	(0.02)	0.58
Discomfort	Mi	1	(0.03)	0	(0)	1	(0.02)	0.58
Discomfort	Mo	1	(0.03)	0	(0)	1	(0.02)	0.58
General discomfort	Mi	19	(0.6)	9	(0.96)	28	(0.69)	0.25
General discomfort	Mo	4	(0.12)	1	(0.1)	5	(0.12)	0.87
Chest tightness	Mi	1	(0.03)	1	(0.1)	2	(0.04)	0.36
Pyrexia	Mi	50	(1.6)	51	(5.44)	101	(2.49)	0.000
Pyrexia	Mo	14	(0.44)	4	(0.42)	18	(0.44)	0.93
Pyrexia	S	1	(0.03)	0	(0)	1	(0.02)	0.58
Decreased thirst	Mi	0	(0)	1	(0.1)	1	(0.02)	0.06
Feeling cold	Mi	0	(0)	1	(0.1)	1	(0.02)	0.07
Musculoskeletal and connective tissue disorders	**Total**	139	(4.45)	51	(5.44)	190	(4.68)	0.20
	**Mi**	98	(3.14)	43	(4.59)	141	(3.47)	0.03
	**Mo**	41	(1.31)	8	(0.85)	49	(1.2)	0.25
	**S**	3	(0.09)	0	(0)	3	(0.07)	0.34
Arthralgia	Mi	29	(0.92)	21	(2.24)	50	(1.23)	0.001
Arthralgia	Mo	10	(0.32)	4	(0.42)	14	(0.34)	0.62
Arthralgia	S	1	(0.03)	0	(0)	1	(0.02)	0.58
Back pain	Mi	5	(0.16)	2	(0.21)	7	(0.17)	0.72
Back pain	Mo	3	(0.09)	0	(0)	3	(0.07)	0.34
Ligament sprain	Mo	0	(0)	1	(0.1)	1	(0.02)	0.07
Muscle spams	Mi	0	(0)	1	(0.1)	1	(0.02)	0.07
Myalgia	Mi	68	(2.17)	24	(2.56)	92	(2.26)	0.48
Myalgia	Mo	31	(0.99)	4	(0.42)	35	(0.86)	0.10
Myalgia	S	2	(0.06)	0	(0)	2	(0.04)	0.43

SOC PT	Severity	AVX	%	AZ	%	Total	%	p-Value
Discomfort in limbs	Mi	1	(0.03)	0	(0)	1	(0.02)	0.58
Ocular disorders	**Total**	1	(0.03)	1	(0.1)	2	(0.04)	0.36
	**Mi**	0	(0)	1	(0.1)	1	(0.02)	0.07
	**Mo**	1	(0.03)	0	(0)	1	(0.02)	0.58
	**S**	0	(0)	0	(0)	0	(0)	
Allergic conjunctivitis	Mo	1	(0.03)	0	(0)	1	(0.02)	0.58
Photopsia	Mi	0	(0)	1	(0.1)	1	(0.02)	0.07
Psychiatric disorders	**Total**	5	(0.16)	3	(0.32)	8	(0.19)	0.33
	**Mi**	5	(0.16)	3	(0.32)	8	(0.19)	0.33
	**Mo**	0	(0)	0	(0)	0	(0)	
	**S**	0	(0)	0	(0)	0	(0)	
Mood changes	Mi	1	(0.03)	0	(0)	1	(0.02)	0.58
Confusional state	Mi	1	(0.03)	0	(0)	1	(0.02)	0.58
Insomnia	Mi	1	(0.03)	2	(0.21)	3	(0.07)	0.07
Irritability	Mi	2	(0.06)	1	(0.1)	3	(0.07)	0.67
Respiratory, thoracic and mediastinal disorders	**Total**	111	(3.55)	24	(2.56)	135	(3.32)	0.13
	**Mi**	88	(2.82)	23	(2.45)	111	(2.73)	0.55
	**Mo**	24	(0.76)	3	(0.32)	27	(0.66)	0.13
	**S**	3	(0.09)	0	(0)	3	(0.07)	0.34
Asthma	Mi	0	(0)	1	(0.1)	1	(0.02)	0.07
Nasal congestion	Mi	12	(0.38)	3	(0.32)	15	(0.36)	0.77
Nasal congestion	Mo	2	(0.06)	0	(0)	2	(0.04)	0.43
Dyspnea	Mi	2	(0.06)	0	(0)	2	(0.04)	0.43
Dyspnea	Mo	0	(0)	1	(0.1)	1	(0.02)	0.07
Dyspnea	S	1	(0.03)	0	(0)	1	(0.02)	0.58
Pharyngolaryngeal pain	Mi	9	(0.28)	2	(0.21)	11	(0.27)	0.69
Pharyngolaryngeal pain	Mo	7	(0.22)	1	(0.1)	8	(0.19)	0.47
Pharyngolaryngeal pain	S	1	(0.03)	0	(0)	1	(0.02)	0.58

SOC PT	Severity	AVX	%	AZ	%	Total	%	p-Value
Hiccups	Mi	1	(0.03)	0	(0)	1	(0.02)	0.58
Allergic rhinitis	Mo	1	(0.03)	0	(0)	1	(0.02)	0.58
Rhinorrhea	Mi	61	(1.95)	14	(1.49)	75	(1.84)	0.36
Rhinorrhea	Mo	15	(0.48)	1	(0.1)	16	(0.39)	0.10
Rhinorrhea	S	1	(0.03)	0	(0)	1	(0.02)	0.58
Cough	Mi	17	(0.54)	7	(0.74)	24	(0.59)	0.47
Cough	Mo	6	(0.19)	1	(0.1)	7	(0.17)	0.58
Cough	S	1	(0.03)	0	(0)	1	(0.02)	0.58
Productive cough	Mi	1	(0.03)	0	(0)	1	(0.02)	0.58
Vascular disorders	**Total**	5	(0.16)	1	(0.1)	6	(0.14)	0.70
	**Mi**	4	(0.12)	1	(0.1)	5	(0.12)	0.87
	**Mo**	1	(0.03)	0	(0)	1	(0.02)	0.58
	**S**	0	(0)	0	(0)	0	(0)	
Flushing	Mi	2	(0.06)	1	(0.1)	3	(0.07)	0.67
Flushing	Mo	1	(0.03)	0	(0)	1	(0.02)	0.58
Hypertensive crisis	Mi	1	(0.03)	0	(0)	1	(0.02)	0.58
Hypertension	Mi	1	(0.03)	0	(0)	1	(0.02)	0.58

SOC: System Organ Class; PT: Preferred term; AE: Adverse Event; Mi: Mild; Mo: Moderate; S: Severe; AVX: AVX/COVID-12; AZ: AZ/ChAdOx-1-S. P-value calculated from comparison of proportions (Z-test).

Table S6. Proportion of subjects affected by vaccine associated adverse events (VAAEs) at 7 days post-immunization.

SOC<br>PT	Severity	AVX	%	AZ	%	Total	%	p-Value
Subjects		3120		936		4056		
Any AE	Total	539	(17.27)	122	(13.03)	661	(16.29)	0.002
	**Mi**	467	(14.96)	115	(12.28)	582	(14.34)	0.04
	**Mo**	90	(2.88)	9	(0.96)	99	(2.44)	0.0008
	**S**	4	(0.12)	0	(0)	4	(0.09)	0.27
Infections and infestations	**Total**	2	(0.06)	0	(0)	2	(0.04)	0.43
	**Mi**	1	(0.03)	0	(0)	1	(0.02)	0.58
	**Mo**	1	(0.03)	0	(0)	1	(0.02)	0.58
	**S**	0	(0)	0	(0)	0	(0)	
Upper respiratory tract infection	Mo	1	(0.03)	0	(0)	1	(0.02)	0.58
Mastitis	Mi	1	(0.03)	0	(0)	1	(0.02)	0.58
Skin and subcutaneous tissue disorders	**Total**	14	(0.44)	4	(0.42)	18	(0.44)	0.93
	**Mi**	9	(0.28)	4	(0.42)	13	(0.32)	0.50
	**Mo**	5	(0.16)	0	(0)	5	(0.12)	0.22
	**S**	0	(0)	0	(0)	0	(0)	
Hyperhidrosis	Mi	0	(0)	1	(0.1)	1	(0.02)	0.07
Papule	Mi	2	(0.06)	1	(0.1)	3	(0.07)	0.67
Pruritus	Mi	7	(0.22)	2	(0.21)	9	(0.22)	0.95
Pruritus	Mo	4	(0.12)	0	(0)	4	(0.09)	0.27
Urticaria	Mo	1	(0.03)	0	(0)	1	(0.02)	0.58
Metabolism and nutrition disorders	**Total**	3	(0.09)	0	(0)	3	(0.07)	0.34
	**Mi**	2	(0.06)	0	(0)	2	(0.04)	0.43
	**Mo**	1	(0.03)	0	(0)	1	(0.02)	0.58
	**S**	0	(0)	0	(0)	0	(0)	
Hypoglycemia	Mo	1	(0.03)	0	(0)	1	(0.02)	0.58
Polydipsia	Mi	2	(0.06)	0	(0)	2	(0.04)	0.43

SOC PT	Severity	AVX	%	AZ	%	Total	%	p-Value
Ear and labyrinth disorders	**Total**	1	(0.03)	1	(0.1)	2	(0.04)	0.36
	**Mi**	1	(0.03)	1	(0.1)	2	(0.04)	0.36
	**Mo**	0	(0)	0	(0)	0	(0)	
	**S**	0	(0)	0	(0)	0	(0)	
Ear pain	Mi	0	(0)	1	(0.1)	1	(0.02)	0.07
Vertigo	Mi	1	(0.03)	0	(0)	1	(0.02)	0.58
Nervous system disorders	**Total**	55	(1.76)	16	(1.7)	71	(1.75)	0.91
	**Mi**	41	(1.31)	16	(1.7)	57	(1.4)	0.36
	**Mo**	14	(0.44)	0	(0)	14	(0.34)	0.04
	**S**	1	(0.03)	0	(0)	1	(0.02)	0.58
Headache	Mi	32	(1.02)	16	(1.7)	48	(1.18)	0.08
Headache	Mo	13	(0.41)	0	(0)	13	(0.32)	0.05
Headache	S	1	(0.03)	0	(0)	1	(0.02)	0.58
Dizziness	Mi	4	(0.12)	0	(0)	4	(0.09)	0.27
Paresthesia	Mi	4	(0.12)	0	(0)	4	(0.09)	0.27
Paresthesia	Mo	1	(0.03)	0	(0)	1	(0.02)	0.58
Drowsiness	Mi	2	(0.06)	0	(0)	2	(0.04)	0.43
Gastrointestinal disorders	**Total**	2	(0.06)	2	(0.21)	4	(0.09)	0.20
	**Mi**	2	(0.06)	2	(0.21)	4	(0.09)	0.20
	**Mo**	0	(0)	0	(0)	0	(0)	
	**S**	0	(0)	0	(0)	0	(0)	
Diarrhea	Mi	2	(0.06)	0	(0)	2	(0.04)	0.43
Nausea	Mi	0	(0)	1	(0.1)	1	(0.02)	0.07
Odynophagia	Mi	0	(0)	1	(0.1)	1	(0.02)	0.07
General disorders and administration site alterations	**Total**	486	(15.57)	114	(12.17)	600	(14.79)	0.01
	**Mi**	421	(13.49)	106	(11.32)	527	(12.99)	0.08
	**Mo**	76	(2.43)	9	(0.96)	85	(2.09)	0.005

SOC PT	Severity	AVX	%	AZ	%	Total	%	p-Value
	**S**	4	(0.12)	0	(0)	4	(0.09)	0.27
Asthenia	Mi	9	(0.28)	1	(0.1)	10	(0.24)	0.32
Asthenia	Mo	1	(0.03)	0	(0)	1	(0.02)	0.58
Pain at the application site	Mi	1	(0.03)	0	(0)	1	(0.02)	0.58
Pain at the injection site	Mi	384	(12.3)	88	(9.4)	472	(11.63)	0.01
Pain at the injection site	Mo	66	(2.11)	9	(0.96)	75	(1.84)	0.02
Pain at the injection site	S	1	(0.03)	0	(0)	1	(0.02)	0.58
Edema at the injection site	Mi	4	(0.12)	1	(0.1)	5	(0.12)	0.87
Edema at the injection site	Mo	5	(0.16)	0	(0)	5	(0.12)	0.22
Erythema at the injection site	Mi	6	(0.19)	3	(0.32)	9	(0.22)	0.46
Erythema at the injection site	Mo	7	(0.22)	0	(0)	7	(0.17)	0.14
Erythema at the injection site	S	1	(0.03)	0	(0)	1	(0.02)	0.58
Chills	Mi	1	(0.03)	3	(0.32)	4	(0.09)	0.01
Chills	Mo	2	(0.06)	0	(0)	2	(0.04)	0.43
Fatigue	Mi	19	(0.6)	9	(0.96)	28	(0.69)	0.25
Fatigue	Mo	2	(0.06)	0	(0)	2	(0.04)	0.43
Fatigue	S	2	(0.06)	0	(0)	2	(0.04)	0.43
Hematoma at the injection site	Mi	1	(0.03)	1	(0.1)	2	(0.04)	0.36
Swelling	Mi	3	(0.09)	2	(0.21)	5	(0.12)	0.36
Induration at the injection site	Mi	2	(0.06)	0	(0)	2	(0.04)	0.43
Inflammation at the injection site	Mi	5	(0.16)	1	(0.1)	6	(0.14)	0.70
Nerve injury at the injection site	Mi	0	(0)	2	(0.21)	2	(0.04)	0.009
Discomfort	Mi	1	(0.03)	0	(0)	1	(0.02)	0.58
General malaise	Mi	5	(0.16)	4	(0.42)	9	(0.22)	0.12
General malaise	Mo	2	(0.06)	0	(0)	2	(0.04)	0.43
Pyrexia	Mi	11	(0.35)	13	(1.38)	24	(0.59)	0.0003
Pyrexia	Mo	3	(0.09)	0	(0)	3	(0.07)	0.34
Decreased thirst	Mi	0	(0)	1	(0.1)	1	(0.02)	0.07

SOC<br>PT	Severity	AVX	%	AZ	%	Total	%	p-Value
Musculoskeletal and connective tissue disorders	**Total**	48	(1.53)	8	(0.85)	56	(1.38)	0.11
	**Mi**	38	(1.21)	7	(0.74)	45	(1.1)	0.22
	**Mo**	9	(0.28)	1	(0.1)	10	(0.24)	0.32
	**S**	1	(0.03)	0	(0)	1	(0.02)	0.58
Arthralgia	Mi	5	(0.16)	5	(0.53)	10	(0.24)	0.04
Arthralgia	Mo	2	(0.06)	0	(0)	2	(0.04)	0.43
Back pain	Mi	2	(0.06)	0	(0)	2	(0.04)	0.43
Pain in limb	Mo	2	(0.06)	0	(0)	2	(0.04)	0.43
Myalgia	Mi	30	(0.96)	3	(0.32)	33	(0.81)	0.05
Myalgia	Mo	6	(0.19)	1	(0.1)	7	(0.17)	0.58
Myalgia	S	1	(0.03)	0	(0)	1	(0.02)	0.58
Discomfort in limbs	Mi	1	(0.03)	0	(0)	1	(0.02)	0.58
Psychiatric disorders	**Total**	2	(0.06)	1	(0.1)	3	(0.07)	0.67
	**Mi**	2	(0.06)	1	(0.1)	3	(0.07)	0.67
	**Mo**	0	(0)	0	(0)	0	(0)	
	**S**	0	(0)	0	(0)	0	(0)	
Irritability	Mi	2	(0.06)	1	(0.1)	3	(0.07)	0.67
Respiratory, thoracic and mediastinal disorders	**Total**	12	(0.38)	2	(0.21)	14	(0.34)	0.43
	**Mi**	10	(0.32)	2	(0.21)	12	(0.29)	0.59
	**Mo**	2	(0.06)	0	(0)	2	(0.04)	0.43
	**S**	0	(0)	0	(0)	0	(0)	
Nasal congestion	Mo	1	(0.03)	0	(0)	1	(0.02)	0.58
Pharyngolaryngeal pain	Mi	1	(0.03)	0	(0)	1	(0.02)	0.58
Pharyngotonsillitis	Mi	1	(0.03)	0	(0)	1	(0.02)	0.58
Rhinorrhea	Mi	8	(0.25)	2	(0.21)	10	(0.24)	0.81
Rhinorrhea	Mo	1	(0.03)	0	(0)	1	(0.02)	0.58
Cough	Mi	0	(0)	1	(0.1)	1	(0.02)	0.07

SOC<br>PT	Severity	AVX	%	AZ	%	Total	%	p-Value
Vascular disorders	**Total**	2	(0.06)	0	(0)	2	(0.04)	0.43
	**Mi**	1	(0.03)	0	(0)	1	(0.02)	0.58
	**Mo**	1	(0.03)	0	(0)	1	(0.02)	0.58
	**S**	0	(0)	0	(0)	0	(0)	
Hypertensive crisis	Mi	1	(0.03)	0	(0)	1	(0.02)	0.58
Hypotension	Mo	1	(0.03)	0	(0)	1	(0.02)	0.58

SOC: System Organ Class; PT: Preferred term; AE: Adverse Event; Mi: Mild; Mo: Moderate; S: Severe; AVX: AVX/COVID-12; AZ: AZ/ChAdOx-1-S. P-value calculated from comparison of proportions (Z-test).

Table S7. Incidence of COVID-19 cases

Subjects in the study	Total 1,417	AVX/COVID-12 705 (49.8)	AZ/ChAdOx-1-S 712 (50.2)
Total follow-up time in days	255,143	127,120	128,023
Average time (SD) in days	180 (31.6)	180.3 (29.6)	179.8 (33.5)
COVID-19 cases (%)[1]	79 (5.5)	37 (5.3)	42 (6.3)
Incidence rate per 1000 days	0.30	0.29	0.32

[1]Log Rank Test: p-value = 0.58. SD = Standard Deviation.
